# A dosimetric comparison of copper and Cerrobend electron inserts

**DOI:** 10.1120/jacmp.v17i5.6282

**Published:** 2016-09-08

**Authors:** Benjamin D. Rusk, Robert L. Carver, John P. Gibbons, Kenneth R. Hogstrom

**Affiliations:** ^1^ Department of Physics and Astronomy Louisiana State University Baton Rouge LA; ^2^ Mary Bird Perkins Cancer Center Baton Rouge LA USA

**Keywords:** electron applicator insert, copper, custom inserts, electron beamcollimation

## Abstract

The purpose of this work was to evaluate differences in dose resulting from the use of copper aperture inserts compared to lead‐alloy (Cerrobend) aperture inserts for electron beam therapy. Specifically, this study examines if copper aperture inserts can be used clinically with the same commissioning data measured using lead‐alloy aperture inserts. The copper inserts were acquired from .decimal, LLC and matching lead‐alloy, Cerrobend inserts were constructed in‐house for 32 combinations of nine square insert field sizes (2×2 to $20\times 20\;{\text{cm}}^{2}$) and five applicator sizes (6×6 to $25\times 25\;{\text{cm}}^{2}$). Percent depth‐dose and off‐axis relative dose profiles were measured using an electron diode in water for select copper and Cerrobend inserts for a subset of applicators ($6\times 6,10\times 10,25\times 25\;{\text{cm}}^{2}$) and energies (6, 12, 20 MeV) at 100 and 110 cm source‐to‐surface distances (SSD) on a Varian Clinac 21EX accelerator. Dose outputs were measured for all field size‐insert combinations and five available energies (6−20 MeV) at 100 cm SSD and for a smaller subset at 110 cm SSD. Using these data, 2D planar absolute dose distributions were generated and compared. Criteria for agreement were ±2% of maximum dose or 1 mm distance‐to‐agreement for 99% of points. A gamma analysis of the beam dosimetry showed 94 of 96 combinations of insert size, applicator, energy, and SSD were within the 2%/1 mm criteria for >99% of points. Outside the field, copper inserts showed less bremsstrahlung dose under the insert compared to Cerrobend (greatest difference was 2.5% at 20 MeV and 100 cm SSD). This effect was most prominent at the highest energies for combinations of large applicators with small field sizes, causing some gamma analysis failures. Inside the field, more electrons scattered from the collimator edge of copper compared to Cerrobend, resulting in an increased dose at the field edge for copper at shallow depths (greatest increase was 1% at 20 MeV and 100 cm SSD). Dose differences decreased as the SSD increased, with no gamma failures at 110 cm SSD. Inserts for field sizes $\geq 6\times 6{\text{cm}}^{2}$ at any energy, or for small fields ($\leq 4\times 4{\text{cm}}^{2}$) at energies <20 MeV, showed dosimetric differences less than 2%/1 mm for more than 99% of points. All areas of comparison criteria failures were from lower out‐of‐field dose under copper inserts due to a reduction in bremsstrahlung production, which is clinically beneficial in reducing dose to healthy tissue outside of the planned treatment volume. All field size‐applicator size‐energy combinations passed 3%/1 mm criteria for 100% of points. Therefore, it should be clinically acceptable to utilize copper insets with dose distributions measured with Cerrobend inserts for treatment planning dose calculations and monitor unit calculations.

PACS number(s): 87.56.jk

## I. INTRODUCTION

Electron therapy treatments typically use patient‐specific devices to conform the lateral beam edges to the physician‐outlined planning treatment volume (PTV). The most common device used is the electron applicator, which attaches below the treatment head and includes three trimmers to further collimate the beam along with a bottom tray for patient‐specific collimation. While photon treatments can use multileaf collimators (MLCs) to achieve patient‐specific collimation, electron MLC prototypes exist but are not commonly available in commercial treatment machines.^1^, ^2^ Instead, electron beams use custom inserts for shaping of the lateral field edges to conform to the PTV while sparing adjacent critical structures and normal tissue.

The most commonly used electron insert, introduced by Powers et al.,^(3)^ is manually fabricated using Lipowitz metal, a lead alloy known by the brand name Cerrobend. Third‐party vendors, such as .decimal, LLC (Sanford, FL), machine custom inserts (99.9% copper) from patients' treatment planning files, with inserts received by the treatment center within 1–3 days of request*. Employing an outside vendor to mill patient‐specific copper inserts offers many advantages. First, it eliminates the safety precautions required for handling Cerrobend, which contains toxic lead and cadmium. Second, milled inserts will be more accurate than manually‐fabricated Cerrobend inserts, which might be important for treatments using abutting fields or having adjacent critical structures. Third, copper is less brittle than Cerrobend, making it less likely to be damaged from repeated use or being dropped. Contrarily, utilizing copper inserts from third‐party vendors can have disadvantages, primarily, shipping time and cost. Also, small modifications of the aperture during clinical setup are more difficult, possibly delaying treatment by a couple of days.

Of clinical concern when using copper inserts is the validity of Cerrobend‐based commissioning data used for treatment planning dose and monitor unit (MU) calculations. Many linear accelerators were commissioned using Cerrobend inserts to determine percent depth‐dose curves (PPD), off‐axis relative dose profiles, output factors, and air gap factors. Differences in these dosimetric data between Cerrobend and copper inserts can decrease the accuracy of the treatment planning system's electron dose calculations. Prior to implementing copper inserts for use in patient treatment, it is prudent to investigate dosimetric differences between copper and Cerrobend electron inserts.

Standard commissioning beam data (PDDs, off‐axis relative dose profiles at various depths, and output factors) were measured for a clinically relevant range of applicator sizes ($6\times 6-25\times 25\;{\text{cm}}^{2}$), insert sizes ($2\times 2-20\times 20\;{\text{cm}}^{2}$), energies (6−20 MeV), and SSDs (100 and 110 cm) for the Varian Clinac 21EX at Mary Bird Perkins Cancer Center (MBPCC). These beam metrics were combined to generate absolute 2D dose distributions, which were evaluated for clinically significant differences caused by the use of copper inserts compared to Cerrobend inserts.

## II. MATERIALS AND METHODS

All electron beam dosimetric data were measured at MBPCC on a Varian Clinac 21EX 4/10 linear accelerator (SN: 1412), following the guidelines from TG‐106.^(4)^ Electron energies available were 6, 9, 12, 16, and 20 MeV ($E_{p,0}=5.95,8.76,12.51,16.36,\;\text{and}19.68\;\text{MeV}$). Applicators used were Varian Type III accessories in sizes of 6×6,10×10,15×15,20×20, and $25\times 25\;{\text{cm}}^{2}$. All measurements (PDDs, off‐axis profiles, and output factors) were relative dose measurements.

### A. Creation of a matching set of copper and Cerrobend inserts

#### A.1 Creating the matching set of inserts

To compare dosimetric differences between copper and Cerrobend inserts for potential clinical use, dose distributions were measured spanning the clinically relevant range of applicators ($6\times 6-25\times 25\;{\text{cm}}^{2}$) and field sizes ($2\times 2-20\times 20\;{\text{cm}}^{2}$ defined at isocenter). Table 1 shows 32 field size‐applicator combinations studied. No dose distribution measurements were taken for the open field sizes, because these field size‐applicator combinations do not require custom inserts.

Inserts were constructed to be of sufficient thickness for the highest energy (20 MeV), a standard procedure for clinical practice, which allows the same insert to be used at all energies (6−20 MeV). The required minimum thickness of lead (t_Pb_) for shielding a 20 MeV electron beam, maximum energy of electron beams ranging from 6−20 MeV, according to AAPM task group 25 is 10 mm.^(5)^ Density scaling from lead ($\rho_{\text{Pb}}=11.34g/{\text{cm}}^{3}$) to Cerrobend ($\rho_{\text{Cerrobend}}=9.38g/{\text{cm}}^{3}$) and copper ($\rho_{\text{Cu}}=8.96g/{\text{cm}}^{3}$) was used to calculate the minimum required thicknesses at 20 MeV of 11.9 cm and 12.5 cm, respectively. Note that the copper inserts must be ∼5.5% thicker than Cerrobend to achieve the same electron shielding.

**Figure 1:**
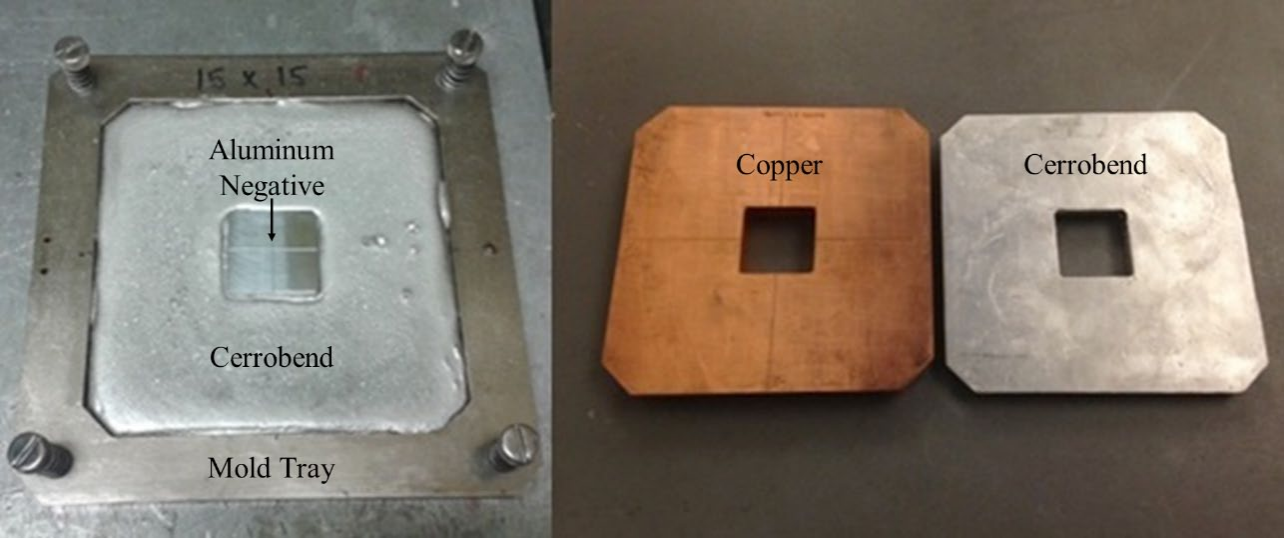
Photo of Cerrobend (left) poured into a $15\times 15\;{\text{cm}}^{2}$ applicator mold to generate an insert formed by a $4\times 4\;{\text{cm}}^{2}$ aluminum negative. The resulting Cerrobend insert (right) alongside its matching copper insert.

The 32 copper inserts were milled by .decimal Inc. along with corresponding aluminum negatives. These negatives were used to mold a matching set of Cerrobend inserts at MBPCC. Figure 1 shows Cerrobend poured into a $15\times 15\;{\text{cm}}^{2}$ applicator mold tray around a centered aluminum negative for a $4\times 4\;{\text{cm}}^{2}$ field, and the resulting Cerrobend insert alongside its matching copper insert.

**Table 1:** Summary of all insert field size‐applicator combinations (X) constructed for dosimetric comparisons. Both copper and Cerrobend inserts were fabricated. Open applicators, requiring no custom insert (N/A), were not compared.

*Field Size (cm^2^)*	*Applicator Size (cm^2^)*				
	6×6	10×10	15×15	20×20	25×25
2×2	X	X	X	X	X
3×3	X	X	X	X	X
4×4	X	X	X	X	X
6×6	N/A	X	X	X	X
8×8		X	X	X	X
10×10		N/A	X	X	X
12×12			X	X	X
15×15			N/A	X	X
20×20				N/A	X

#### A.2 Quality assurance of matching electron inserts

Thickness measurements were taken using a digital caliper with a 0.002 cm precision. In addition, the square field size in the X and Y direction of each insert was measured using a digital caliper with 0.002 cm precision, except for the largest field size ($20\times 20\;{\text{cm}}^{2}$), which was measured using a ruler with 0.5 mm precision due to the size limitations of the digital caliper.

The average measured thickness of all the copper inserts was 14.80 mm, with a standard deviation (SD) of 0.07 mm. The average measured thickness of all Cerrobend inserts was 12.46 mm, with a standard deviation of 0.89 mm. The electron transmitted dose ($D-D_{\text{bremsstrahlung}}$) with shielding was less than 2% of maximum dose without shielding in the 6‐20 MeV energy range, with residual levels being due to bremsstrahlung produced photons, rather than electron transmission. Therefore the difference in electron transmission through the copper and Cerrobend inserts was expected to be negligible. A complete table of all copper and Cerrobend insert thicknesses can be found in Rusk's Master's thesis.^(6)^

The average difference between the X and Y field size for every insert was 0.07 mm with a standard deviation of 0.08 mm for copper and 0.15 mm with a standard deviation of 0.13 mm for Cerrobend. The mean of the X and Y measurements for each insert formed the average field size. Comparing the average field sizes between matching copper and Cerrobend inserts, the average difference (Cerrobend minus copper) in field sizes was 0.15 mm, with a standard deviation of 0.10 mm and a maximum difference of 0.33 mm. These differences are negligible (<0.5mm), so the copper and Cerrobend inserts were treated as having the same field sizes.

### B. Measurement of dosimetric data

#### B.1 Measurement equipment

Dose measurements were made using a p‐type electron dosimetry diode detector (IBA EFD^3G^, #300‐605; Iba Dosimetry, Schwarzenbruck, Germany) with an active volume diameter of 2 mm and thickness of 0.06 mm. Silicon‐diode detectors measure ionization in the active region of the diode, where it is assumed that ionization is proportional to dose (i.e., correction factors are energy independent). These detectors can be used to accurately measure relative dose distributions for high energy electron beams.^7^, ^8^

The diode was connected to the beam scanning main control unit (MCU), which contained an internal electrometer for PDD and off‐axis relative dose profile measurements. For output measurements the diode was connected to an external calibrated electrometer. This arrangement allowed the use of the 2D scanning motors to precisely position the diode at depths for output readings using the MCU software. Since the diode and water phantom setup remained unchanged between scans and output measurements, the setup also ensured consistency in the geometry for all measurements.

All PDDs, off‐axis relative dose profiles, and output measurements (100 cm SSD) were taken in a RFA‐200 water phantom 2D scanning tank using OmniPro scanning software (Iba Dosimetry). The phantom was leveled in all directions to assure scanning would be aligned with the electron beam. The diode was placed near the center of the phantom and the couch adjusted laterally and longitudinally to align the diode with the center of the light field from the linear accelerator. The couch was adjusted vertically to the desired SSD using mechanical distance indicators. Periodically the SSD was verified by using the optical distance indicator (ODI). The flat entry surface of the diode was then visually set even to the water surface, and its position was zeroed in the scanning software by adjusting for the known effective measurement location. Because of the long duration of scanning, care was taken to maintain a constant water level. The water level was checked regularly throughout the day and water added to compensate for any evaporation.

Quality assurance (QA) of the measuring apparatus was done daily to ensure the mechanical stability of the scanning equipment as well as energy stability of the linear accelerator. QA for the mechanical scanning equipment was performed by taking three consecutive PDD scans of the 9 MeV beam at least once per day. Verifying that the $R_{50}$ values of the three scans were within a tolerance of ±0.05cm ensured that the mechanical components of the 2D scanning phantom were operating properly.

QA for the stability of the measurement equipment was performed by measuring a 9 MeV PDD using the open field size in whichever applicator was being used for the measurements that day. The beginning‐of‐day and end‐of‐day PDDs were then compared to ensure that the $R_{50}$ values were within a tolerance of ±0.1cm. This ensured that the beam scanner was aligned properly and that the diode and electrometer were functioning properly. The 9 MeV beam was chosen because of its sharp falloff in the PDD, which facilitates $R_{50}$ measurement while also having greater depth of penetration than the 6 MeV beam. The QA procedure also gave confidence that the energy of the 9 MeV beam had not changed throughout the day. However, other energies were not evaluated because measurements comparing copper and Cerrobend were always done sequentially.

#### B.2 Measurement subsets

Measurement subsets were chosen to span the range of clinical combinations of energy, applicator, field size, and SSD. PDD curves and outputs at 100 cm SSD were measured for all five available energies (6, 9, 12, 16, and 20 MeV) for all field size‐applicator combinations (Table 1). Off‐axis relative dose profiles were measured for copper and Cerrobend inserts using three energies (6, 12, and 20 MeV) at 100 cm and 110 cm SSD for the field size‐applicator combinations shown in Table 2. This subset was also used for measuring PDD curves and outputs at 110 cm SSD. This subset was chosen to sample field sizes using the smallest, middle, and largest sized applicators available on the Varian machine. Most clinical electron beam treatments use an energy, SSD, and field size/applicator size geometry in the range spanned by this subset.

**Table 2:** Measurement subset (X) for off‐axis relative dose profile measurements using energies 6, 12, and 20 MeV at 100 cm and 110 cm SSD. This measurement subset was also used for PDD and output measurements at 110 cm SSD.

*Field Size (cm^2^)*	*Applicator Size (cm^2^)*				
	6×6	10×10	15×15	20×20	25×25
2×2	X		X		X
3×3	X				X
4×4	X		X		X
6×6					X
8×8			X		X
10×10					X
12×12			X		X
15×15					X
20×20					X

#### B.3 Percent depth‐dose curves

PDD curves were measured using the OmniPro scanning software with a 1 mm step size and low scan speed in precision mode. All beam scans followed the guidelines described by TG‐255^(9)^ and TG‐51.^(10)^ PDD scans were made from deeper to shallower depths, beginning at depths of 8, 12, and 14 cm for energies of 6, 12, and 20 MeV, respectively. These PDDs were used to compare beam metrics and to create isodose plots for evaluation and comparison. In addition, a more extensive subset was used for measuring PDD curves and outputs at 100 cm SSD. These measurements were taken at all five available energies (6, 9, 12, 16, and 20 MeV) for all field size‐applicator combinations.

#### B.4 Off‐axis relative dose profiles

Off‐axis relative dose profiles were measured with the OmniPro scanning software using a step size of 2 mm and a low scan speed in precision mode. The off‐axis relative dose profiles were measured immediately after the PDDs for each insert. A scan consisted of off‐axis profiles measured at multiple depths beginning 0.5 cm below the surface of the water. The number and depths of the off‐axis relative dose profiles were selected for each energy to acquire data in the high gradient regions and to cover the entire practical range of the beam. Eleven off‐axis profiles (0.5–5.0 cm depth) were measured for 6 MeV beams, 15 profiles (0.5–8.0 cm depth) for 12 MeV beams, and 22 (0.5–12.0 cm depth) profiles for 20 MeV beams.^(6)^ For all off‐axis profile measurements, scanning spanned a 4 cm extension to the diverging field edge of the deepest profile. These measurements allowed significant data for evaluation of out‐of‐field dosimetry. The off‐axis data were combined with PDDs to construct a full 2D dose grid from which isodose curves could be plotted.

#### B.5 Output correction factors

The output correction factor (OCF) is defined (Eq. 1) as the ratio of the average copper output reading at the $R_{100}$ for copper divided by the average Cerrobend output reading at the $R_{100}$ for Cerrobend for a particular energy, applicator, field size, and SSD. The ratio of outputs equals the ratio of electrometer readings for these measurements. Electron beam relative outputs were measured at 100 cm SSD using a 2D water phantom and at 110 cm SSD using a 1D water phantom (DoseView 1D, Standard Imaging Inc., Middleton WI). Relative dose measurements were taken at $R_{100}$ using an external electrometer (CNMC Model 206 dosimetry electrometer) and the same electron diode used for the PDDs and off‐axis relative dose profile measurements. The internal electrometer of the IBA MCU was not designed for measuring the output of a single diode. Therefore, a cable connecting the diode to the external electrometer outside of the vault allowed for easy transition from the MCU to an external electrometer.

The electron inserts were aligned with the central axis using the etchings for copper inserts and a ruler for the Cerrobend inserts. The diode was centered using the linear accelerator's crosshairs. Initial off‐axis profile scans were taken with each new insert to check this centering. Each centering profile was taken in‐plane at a depth of 1 cm. Inserts were considered properly centered if the measured off‐axis profile centers were <0.05 cm from the beam center, with couch adjustments used to align the diode with the beam center as necessary. These measurements ensured that the diode was aligned with the radiation central axis.

After ensuring the diode was centered, a PDD was measured to determine $R_{100}$. Using OmniPro, the diode was repositioned to $R_{100}$. The diode detector was then disconnected from the MCU and connected to the external electrometer. Three electrometer readings were recorded, each with the machine delivering 200 monitor units (MUs), and then averaged. Cerrobend and copper insert outputs were measured at the $R_{100}$ corresponding to Cerrobend. This process was repeated for all five beam energies for a single Cerrobend insert, and then repeated for the matching copper insert immediately afterwards. Consecutively measuring the matching Cerrobend and copper inserts resulted in less than 30 min between measurement sets of the same energy and insert size for the two materials.

There was close agreement between $R_{100}$ values for Cerrobend and copper inserts, with an average difference of less than 0.1 cm. The differences in $R_{100}$ locations all resulted in PDD corrections of less than 0.1%. As such, the OCFs were calculated using Eq. (1) without PDD corrections to the copper output measurement.

(1) $$\text{OCF}(E,\text{Appl},FS,SSD)=\frac{O^{Cu}(E,\text{Appl},FS,\text{SSD})}{O^{\text{Cerrobend}}(E,\text{Appl},FS,\text{SSD})}$$

The uncertainty for the OCF calculations was estimated to be ±0.001.^(6)^ The OCFs were computed at 100 and 110 cm SSD for the measurement subsets shown in 1, 2, respectively.

### C. Comparison of beam dosimetry

#### C.1 Data processing

Prior to the comparison of absolute beam dosimetry, postprocessing of the raw data in OmniPro was done in the following order. 1) PDD data were normalized to 100% at the depth of maximum dose ($R_{100}$); 2) off‐axis relative dose profiles were centered; 3) off‐axis profiles were symmetrized using the mean value from both sides; and 4) profiles were renormalized to the central axis value (from the PDD). No smoothing filters were applied to any scan.

#### C.2 Creation of absolute 2D dose distributions

The OCFs were used to scale the relative dose distributions for copper inserts to create “absolute” dose distributions. Absolute 2D dose distributions are the relative dose distributions normalized such that 100% corresponds to the central‐axis dose measurement at $R_{100}$ for Cerrobend ((2), (3)):

(2) $$D_{\text{Absolute}}^{\text{Copper}}(x,z)=D_{\text{Relative}}^{\text{Copper}}(x,z)\times \text{OCF}$$

(3) $$D_{\text{Absolute}}^{\text{Cerrobend}}(x,z)=D_{\text{Relative}}^{\text{Cerrobend}}(x,z)$$

Absolute 2D dose distributions for matching copper and Cerrobend inserts under the same measurement conditions (i.e., applicator, field size, energy, SSD) were overlaid with isodose lines plotted for visual interpretation of the dose distributions.

#### C.3 Comparison criteria

To implement copper inserts clinically without recommissioning (i.e., using dosimetry data previously measured with Cerrobend inserts), the dosimetric differences between copper and Cerrobend inserts must be clinically acceptable. Annual quality assurance procedures from TG‐40^(11)^ and TG‐142^(12)^ recommend measuring a subset of the commissioning data and comparing to the baseline data to determine dosimetric accuracy, as was done in this study. The dosimetric tolerances described by these Task Group reports were used as comparison criteria.

Cerrobend and copper insert dosimetry data were compared quantitatively on dose distributions with the same delivery geometry. Using a 2%/1 mm criteria, the superimposed dose distributions were checked at each point for agreement to within 2% of the central axis maximum dose for Cerrobend (i.e., the 100% point) or a point which agrees within a radius of 1 mm in the dose measurement plane. The percentage of points passing the criteria was recorded. Any comparison containing failing points were reanalyzed using a 3%/1 mm criteria.

The dosimetric criteria of ±2% of maximum dose or ±1mm distance to agreement (DTA), consistent with TG‐142, was used as a metric for output factors and beam quality. Analysis was performed using the percent of maximum dose difference rather than simply the percent difference because of the greater clinical significance of percent of maximum dose.

## III. RESULTS AND DISCUSSION

### A. Measurement of dosimetric data

#### A.1 Percent depth‐dose curves at 100 cm SSD

**Figure 2:**
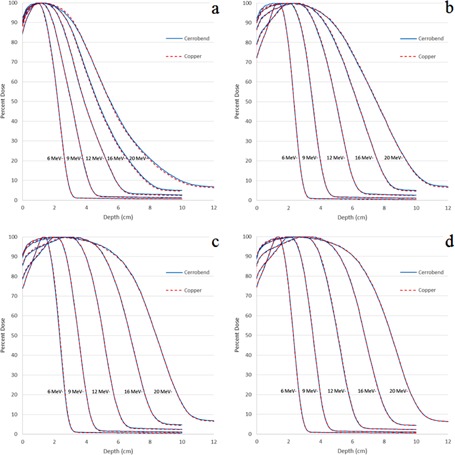
Comparison plots of PDDs for different field sizes in the $25\times 25{\text{cm}}^{2}$ applicator at 100 cm SSD. Field sizes shown are (a) $2\times 2\;{\text{cm}}^{2}$, (b) $4\times 4\;{\text{cm}}^{2}$, (c) $12\times 12\;{\text{cm}}^{2}$, and (d) $20\times 20\;{\text{cm}}^{2}$.

Percent depth‐dose (PDD) curves at 100 cm SSD were measured for all field size ($2\times 2-20\times 20\;{\text{cm}}^{2}$) and applicator ($6\times 6-25\times 25\;{\text{cm}}^{2}$) combinations shown in Table 1 for all energies 6−20 MeV using both Cerrobend and copper inserts. Measured percent depth‐dose curve comparisons between copper and Cerrobend at 100 cm SSD are shown for all energies and a sampling of field sizes ($2\times 2-20\times 20\;{\text{cm}}^{2}$) in the $25\times 25\;{\text{cm}}^{2}$ applicator (Fig. 2). The most notable differences were at deeper depths for the smaller field sizes (2×2 and $4\times 4\;{\text{cm}}^{2}$), possibly due to decreased bremsstrahlung production in the copper insert. Overall, PDDs showed negligible (<1%/1 mm) differences between copper and Cerrobend for the entire PDD curves (surface, peak, and fall‐off regions).

Percent depth‐dose metrics were compared at 100 cm SSD for the 160 pairs of PDDs arising from the 32 field size and applicator size combinations, five energies, and two materials. Metrics included $R_{50},R_{90}$, and $R_{80-20}$. The dose at 1.0 cm depth ($D_{1.0}$) was also compared between the inserts to examine dose differences at shallow depths. The differences in each of these metrics between matching copper and Cerrobend inserts at the same energy were calculated by taking the Cerrobend value minus the copper value.

All PDD metric comparisons averaged over all field sizes showed negligible differences (<0.1cm) between copper and Cerrobend inserts. The maximum differences for $R_{50},R_{90}$, and $R_{80-20}$ were 0.07 cm, 0.13 cm, and 0.08 cm, respectively. The maximum difference in $D_{1.0}$ between the two materials was −0.90% of central axis dose maximum. All 100 cm PDD plots and metric data can be found in Appendix A of Rusk.^(6)^

#### A.2 Percent depth‐dose curves at 110 cm SSD

**Figure 3:**
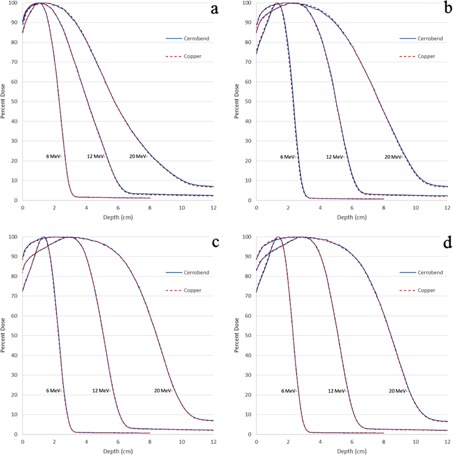
Comparison plots of PDDs for different field sizes in the $25\times 25{\text{cm}}^{2}$ applicator at 110 cm SSD. Field sizes shown are (a) $2\times 2\;{\text{cm}}^{2}$, (b) $4\times 4\;{\text{cm}}^{2}$, (c) $12\times 12\;{\text{cm}}^{2}$ and (d) $20\times 20\;{\text{cm}}^{2}$.

Percent depth‐dose curves at 110 cm SSD were measured for the subset of field size‐applicator combinations listed in Table 2 for energies of 6 MeV, 12 MeV, and 20 MeV. The PDDs showed negligible (<1%/1mm) differences between copper and Cerrobend for the entire PDD curves (surface, peak, and fall‐off regions). Measured PDD comparisons between copper and Cerrobend at 110 cm SSD are shown in Fig. 3 for all energies and a sampling of field sizes in the $25\times 25\;{\text{cm}}^{2}$ applicator.

The same percent depth‐dose metrics were compared at 110 cm SSD as 100 cm SSD for the 48 pairs of PDDs arising from 16 field size/applicator size combinations, 3 energies, and 2 materials. All PDD metric comparisons showed negligible differences between copper and Cerrobend inserts. The maximum differences for $R_{50},R_{90}$, and $R_{80-20}$ were 0.01 cm, 0.02 cm, and 0.09 cm, respectively. The maximum difference in $D_{1.0}$ between the two materials was 0.80% of central axis dose maximum. All 110 cm PDD plots and metric data can be found in Appendix B of Rusk.^(6)^

#### A.3 Central axis photon dose

Percent depth‐dose curves showed little variation between copper and Cerrobend at depths smaller than the practical range ($R_{P}$) for all energies and field size combinations. However, the bremsstrahlung dose ($D_{x}$) in the tail region of the PDDs did show consistent differences (≥0.1) at energies 12 MeV and higher. As energy and applicator size increased and as field size decreased, the difference in $D_{x}$ (Cerrobend minus copper) increased, as shown in Table 3.

Bremsstrahlung production increased with the surface area of the insert material in the beam. A $2\times 2\;{\text{cm}}^{2}$ field size in a $25\times 25\;{\text{cm}}^{2}$ applicator has 19.4 times more insert material being struck by the electron beam than a $2\times 2\;{\text{cm}}^{2}$ field size in a $6\times 6\;{\text{cm}}^{2}$ applicator. This increased bremsstrahlung production to the center of the field resulted in up to a 0.5% of maximum dose increase in central axis dose for Cerrobend inserts compared to copper inserts This effect was greatest when the field is small ($2\times 2\;{\text{cm}}^{2}$), the applicator was large ($25\times 25\;{\text{cm}}^{2}$), and the energy was high (20 MeV).

**Table 3:** Central‐axis photon dose difference (Cerrobend ‐ copper) at a depth of Rp+1. Values presented are a percentage of the central‐axis maximum dose. Generally, as the applicator size and energy increase, the difference increases. In addition as the field size increases, the difference decreases.

$\Delta D_{x}(\%D_{\text{max}})(\text{Cerr}-\text{Cu})$	*6 MeV*			*12 MeV*			*20 MeV*		
*Field Size (cm^2^)*	*Applicator (cm^2^)*			*Applicator (cm^2^)*			*Applicator (cm^2^)*		
	6×6	20×20	25×25	6×6	20×20	25×25	6×6	20×20	25×25
2×2	0.0%	0.0%	0.0%	0.0%	0.2%	0.3%	0.0%	0.5%	0.5%
4×4	−0.1%	0.0%	0.0%	0.0%	0.1%	0.2%	0.0%	0.1%	0.4%
12×12	N/A	0.0%	0.1%	N/A	0.1%	0.1%	N/A	0.2%	0.3%

#### A.4 Off‐axis relative dose profiles

**Figure 4:**
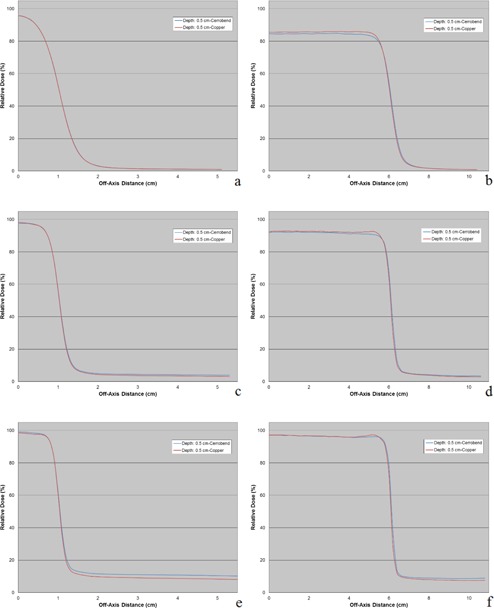
Off‐axis relative dose profile measurements for copper and Cerrobend inserts of $2\times 2\;{\text{cm}}^{2}$ field size (left) and $12\times 12\;{\text{cm}}^{2}$ field size (right) for the $25\times 25\;{\text{cm}}^{2}$ applicator at 100 cm SSD (depth = 0.5 cm). Energies of 6 MeV, 12 MeV, and 20 MeV are shown for the $2\times 2\;{\text{cm}}^{2}$ field size ((a), (c), and (e), respectively) and the $12\times 12\;{\text{cm}}^{2}$ field size ((b), (d), (f), respectively). Profiles are normalized to the central‐axis maximum dose maxima ($D_{\text{max}}$) at $R_{100}$.

Measured off‐axis relative dose profiles from copper and Cerrobend inserts had good overall agreement for all energies, SSDs, and insert/applicator combinations with differences predominantly <2% of maximum dose. Off‐axis relative dose profiles showed the greatest differences between copper and Cerrobend at the shallowest measured depth of 0.5 cm. The observed dosimetric differences were located near the beam edge inside the field and in out‐of‐field regions, as shown in Fig. 4. This higher out‐of‐field dose from Cerrobend inserts as compared to copper inserts was attributed to the relative decrease in the amount of bremsstrahlung production in copper, discussed.

Off‐axis relative dose profiles showed higher doses inside the beam edges for copper inserts than for Cerrobend inserts at depths of less than ∼2cm, being more prominent with higher energy beams and shallower depths. At depths past ∼2cm, no differences in the dose inside the beam edges between the two materials were observed. These beam‐edge horns were attributed to electrons scattering from the collimator edge, illustrated by an inverse relationship between the absorbed dose from scatter and the density of the collimating material.^13^, ^14^ The lower density of copper compared to Cerrobend caused more electron scatter from the insert edge, and thus a higher dose at the field edge. These edge effects never exceeded 2% of central‐axis maximum dose.

Dosimetric differences were also seen in the out‐of‐field region shielded by the insert at off‐axis distances greater than ∼2cm outside the beam edge. While the 6 MeV energy showed no distinct differences in out‐of‐field dose, off‐axis profiles showed higher out‐of‐field doses for Cerrobend than copper at 12 MeV and 20 MeV, with these differences most noticeable at 20 MeV where they sometimes exceeded the ±2% tolerance. As the depth of the measured profile increased this difference became less pronounced.

This higher out‐of‐field dose from Cerrobend inserts as compared to copper inserts was attributed to the relative decrease in the amount of bremsstrahlung production in copper, discussed previously. Out‐of‐field doses from Cerrobend showed dose increases >2% of maximum dose compared to copper for some inserts. This effect gives copper inserts a potential clinical advantage by lowering the risk of secondary cancers through reducing the out‐of‐field dose.

#### A.5 Output correction factors

OCFs (copper/Cerrobend) at 100 cm SSD ranged from 0.983 ($3\times 3\;{\text{cm}}^{2}$ field size, $25\times 25\;{\text{cm}}^{2}$ applicator, 16 MeV) to 1.009 ($6\times 6\;{\text{cm}}^{2}$ field size, $10\times 10\;{\text{cm}}^{2}$ applicator, 20 MeV). All OCFs at 100 cm SSD were within ±2% of unity. The average OCF was 0.999, with Cerrobend having a 0.1% average higher output than copper, as shown in Table 4. Table 5 shows a subset of OCFs covering the clinical range of energies, field sizes, and applicators. Measured average OCFs were 0.999 at 6 MeV, 9 MeV, and 12 MeV, while the average OCF was 0.998 at 16 MeV and 20 MeV. The slightly higher average outputs from Cerrobend inserts compared to copper inserts could be caused by the greater bremsstrahlung production in Cerrobend, especially at higher energies. A table of all OCF for 100 SSD cm can be found in Appendix C of Rusk.^(6)^

OCFs at 110 cm SSD ranged from 0.990 ($2\times 2\;{\text{cm}}^{2}$ field size, $25\times 25\;{\text{cm}}^{2}$ applicator, 6 MeV, $3\times 3\;{\text{cm}}^{2}$ field size, $25\times 25\;{\text{cm}}^{2}$ applicator, 20 MeV, and $4\times 4\;{\text{cm}}^{2}$ field size, $25\times 25\;{\text{cm}}^{2}$ applicator, 20 MeV) to 1.006 ($4\times 4\;{\text{cm}}^{2}$ field size, $15\times 15\;{\text{cm}}^{2}$ applicator, 6 MeV and $8\times 8\;{\text{cm}}^{2}$ field size/$15\times 15\;{\text{cm}}^{2}$ applicator, 6 MeV). All OCFs at 110 cm SSD were within ±1% of unity. For 110 cm SSD, the average OCF over all energies was 0.999, with 6 MeV having the largest average (1.001) and 20 MeV the smallest average (0.997), as shown in Table 6. Table 7 shows a subset of OCFs covering the clinical range of energies, field sizes, and applicators. The slightly higher average outputs from Cerrobend inserts compared to copper inserts at 20 MeV might be caused by the greater bremsstrahlung production in Cerrobend at higher energies. A table of all OCF for 110 SSD cm can be found in Appendix D of Rusk.^(6)^

**Table 4:** Minimum and maximum output correction factors at each energy and for all energies measured at 100 cm SSD.

*Energy*	*Minimum OCF*	*Maximum OCF*
6 MeV	0.992	1.008
9 MeV	0.992	1.006
12 MeV	0.988	1.005
16 MeV	0.983	1.005
20 MeV	0.986	1.009
All Energies	0.983	1.009

**Table 5:** A subset of the OCFs covering the range of energies, field sizes, and applicators measured at 100 cm SSD. “N/A” depicts physically impossible setups.

OCF	*6 MeV*			*12 MeV*			*20 MeV*		
*Field Size (cm^2^)*	*Applicator (cm^2^)*			*Applicator (cm^2^)*			*Applicator (cm^2^)*		
	6×6	15×15	25×25	6×6	15×15	25×25	6×6	15×15	25×25
2×2	1.000	0.993	0.992	1.005	0.998	1.000	1.003	0.991	0.991
3×3	1.007	1.002	0.995	1.005	1.000	0.988	1.007	0.995	0.986
4×4	1.004	1.008	0.999	1.004	1.003	0.994	1.007	0.997	0.988
6×6	N/A	1.002	0.997	N/A	1.001	0.995	N/A	1.003	0.994
8×8	N/A	0.999	0.997	N/A	1.000	0.995	N/A	1.003	0.994
10×10	N/A	0.998	0.998	N/A	0.999	0.995	N/A	1.001	0.996
12×12	N/A	0.996	0.997	N/A	0.996	0.995	N/A	1.000	0.997
15×15	N/A	N/A	0.995	N/A	N/A	0.994	N/A	N/A	0.995
20×20	N/A	N/A	0.996	N/A	N/A	0.995	N/A	N/A	0.995

**Table 6:** Minimum and maximum output correction factors at each energy and for all energies measured at 110 cm SSD.

*Energy*	*Minimum OCF*	*Maximum OCF*
6 MeV	0.990	1.006
12 MeV	0.994	1.005
20 MeV	0.990	1.003
All Energies	0.990	1.006

**Table 7:** A subset of the OCFs covering the range of energies, field sizes, and applicators measured at 110 cm SSD. “N/A” depicts physically impossible setups, whereas dashes denote physically available combinations that were not measured.

*OCF*	*6 MeV*			*12 MeV*			*20 MeV*		
*Field Size (cm^2^)*	*Applicator (cm^2^)*			*Applicator (cm^2^)*			*Applicator (cm^2^)*		
	6×6	15×15	25×25	6×6	15×15	25×25	6×6	15×15	25×25
2×2	1.005	0.993	0.990	0.994	0.996	0.997	1.002	0.997	0.993
4×4	1.004	1.006	1.003	1.004	1.005	1.002	1.001	1.000	0.990
6×6	N/A	‐	1.001	N/A	‐	0.998	N/A	‐	0.992
8×8	N/A	1.006	1.004	N/A	1.005	1.002	N/A	1.002	0.995
10×10	N/A	‐	1.004	N/A	‐	1.002	N/A	‐	0.997
12×12	N/A	1.000	1.002	N/A	0.999	1.000	N/A	1.000	0.997
15×15	N/A	N/A	1.000	N/A	N/A	0.999	N/A	N/A	0.999
20×20	N/A	N/A	1.000	N/A	N/A	1.000	N/A	N/A	1.000

### B. Comparison of beam dosimetry

#### B.1 Analysis of isodose plots at 100 cm SSD

**Figure 5:**
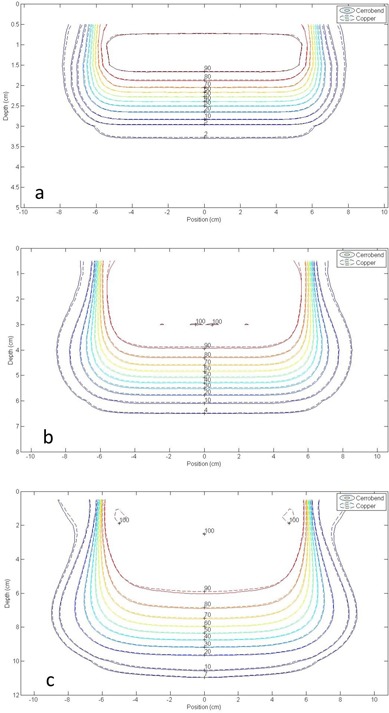
Absolute isodose comparison between Cerrobend (solid lines) and copper (dashed lines) for a $12\times 12\;{\text{cm}}^{2}$ insert in a $15\times 15\;{\text{cm}}^{2}$ applicator at (a) 6 MeV, (b) 12 MeV, and (c) 20 MeV and 100 cm SSD. All points passed the 2%/1 mm criteria. The OCF were 0.996, 0.996, and 1.000, respectively.

**Figure 6:**
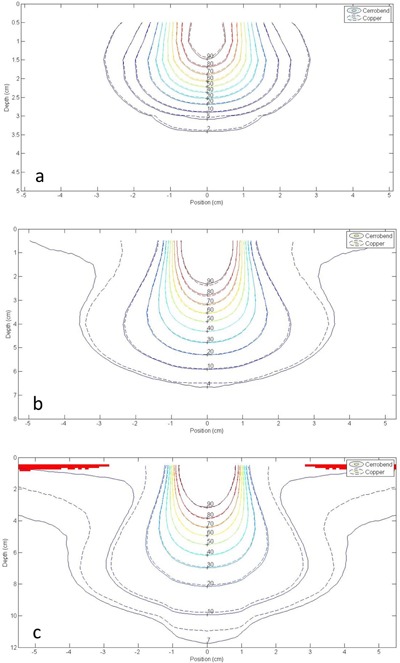
Absolute isodose comparison between Cerrobend (solid lines) and copper (dashed lines) for a $2\times 2\;{\text{cm}}^{2}$ insert in a $25\times 25\;{\text{cm}}^{2}$ applicator at (a) 6 MeV, (b) 12 MeV, and (c) 20 MeV and 100 cm SSD. The red pixels on the isodose plot mark points which failed the 2%/1 mm criteria (98.90% of points passed criteria). The OCF were 0.992, 1.001, and 0.991,respectively.

Figure 5 shows isodose comparisons between copper and Cerrobend dose distributions for the $2\times 2\;{\text{cm}}^{2}$ field in the $25\times 25\;{\text{cm}}^{2}$ applicator for the 6, 12, and 20 MeV beams. Figure 6 shows isodose comparisons between copper and Cerrobend dose distributions for the $12\times 12\;{\text{cm}}^{2}$ field in the $15\times 15\;{\text{cm}}^{2}$ applicator for the 6, 12, and 20 MeV beams. Isodose plots for all measured dose distribution comparisons at 100 cm SSD can be found in Appendix E of Rusk.^(6)^

Of the 48 total combinations of field size, applicator size, and energy, 43 (90%) passed the 2%/1 mm criteria for 100% of points. For 46 of 48 (96%) combinations, ≥99% of points passed the 2%/1 mm criteria; the two failing combinations were the $2\times 2\;{\text{cm}}^{2}$ field size (98.90% passing) and the $4\times 4\;{\text{cm}}^{2}$ field size (98.35% passing) both in the $25\times 25\;{\text{cm}}^{2}$ applicator at 20 MeV. The other three combinations showing point failures were the next three largest field sizes in the $25\times 25\;{\text{cm}}^{2}$ applicator at 20 MeV: $6\times 6\;{\text{cm}}^{2}$ field size (99.44% passing), $8\times 8\;{\text{cm}}^{2}$ field size (99.92% passing), and the $10\times 10\;{\text{cm}}^{2}$ field size (99.67% passing).

At 20 MeV, the additional out‐of‐field bremsstrahlung dose produced in the Cerrobend inserts compared to the copper inserts caused the observed failures in the 2%/1 mm criteria. The off‐axis dose due to bremsstrahlung photons is higher for the Cerrobend insert than the copper insert by a maximum of 2.2% at 0.5 cm depth in this off‐axis region. A detailed analysis of the off‐axis bremsstrahlung differences can be found in Rusk.^(6)^

All criteria failures occurred out‐of‐field due to the lower bremsstrahlung dose from copper inserts compared to Cerrobend inserts, a difference which reduces dose to healthy tissue and is clinically beneficial. Clinically, an insert is created using the smallest possible applicator for the given field size. The inserts which registered criteria failures in this study were small field size‐large applicator combinations that are unlikely to be used for patient treatment.

The increased scatter through the edge of copper inserts compared to Cerrobend inserts did not result in criteria failure for any of the isodose comparisons. While this increased scatter was noticeable in some off‐axis relative dose profiles at shallow depths, the increased scatter did not impact the isodose comparisons. Maximum differences in the beam edge region for copper inserts were less than the 2%/1 mm criteria when compared to Cerrobend inserts.

#### B.2 Analysis of isodose plots at 110 cm SSD

Isodose comparisons between the copper and Cerrobend inserts using the 2%/1 mm DTA criteria at 110 cm SSD showed all 48 of 48 comparisons passing the criteria for 100% of points. 7, 8 show representative samples of the best and worst agreement, respectively, between the Cerrobend and copper dose distributions. The increased out‐of‐field dose from the Cerrobend inserts at extended SSD as compared to the copper inserts was still apparent. However, the differences between the out‐of‐field doses were less than those observed at 100 cm SSD. Isodose plots for all measured dose distribution comparisons at 110 cm SSD can be found in Appendix F of Rusk.^(6)^

**Figure 7:**
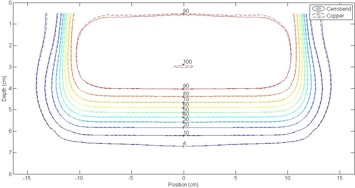
Absolute isodose comparison between Cerrobend (solid lines) and copper (dashed lines) for a $20\times 20\;{\text{cm}}^{2}$ insert in a $25\times 25\;{\text{cm}}^{2}$ applicator at 12 MeV and 110 cm SSD. All points passed the 2%/1 mm criteria. The OCF was 1.00.

**Figure 8:**
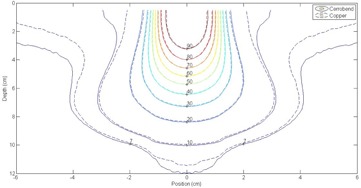
Absolute isodose comparison between Cerrobend (solid lines) and copper (dashed lines) for a $2\times 2\;{\text{cm}}^{2}$ insert in a $25\times 25\;{\text{cm}}^{2}$ applicator at 20 MeV and 110 cm SSD. All points passed the 2%/1 mm criteria. The OCF was 0.993.

## IV. CONCLUSIONS AND RECOMMENDATIONS

Using custom milled copper inserts for electron beam therapy planned with standard commissioning data measured on Cerrobend inserts should result in minimal absolute dosimetric differences (≥99% of points within ±2% of $D_{\text{max}}$ or 1 mm DTA) for standard clinical field sizes ($2\times 2\;{\text{cm}}^{2}$ to $20\times 20\;{\text{cm}}^{2}$), applicators ($6\times 6\;{\text{cm}}^{2}$ to $25\times 25\;{\text{cm}}^{2}$), and energies (6 MeV to 20 MeV). At 100 cm SSD, all dosimetric comparisons of copper and Cerrobend inserts passed a 3%/1 mm criteria for 100% of the area. At 100 cm SSD, comparisons of the absolute dosimetric difference between copper and Cerrobend inserts showed 100% of the area within 2%/1 mm agreement for all field sizes at energies of 6 and 12 MeV. Only copper and Cerrobend small field inserts ($2\times 2\;{\text{cm}}^{2}$ and $4\times 4\;{\text{cm}}^{2}$) in the $25\times 25\;{\text{cm}}^{2}$ applicator at 20 MeV resulted in less than 99% of the area passing the 2%/1 mm comparison criteria, with the worst case being a 98.35% passing rate for the $4\times 4\;{\text{cm}}^{2}$ field in the $25\times 25\;{\text{cm}}^{2}$ applicator. At 110 cm SSD, all dosimetric comparisons of copper and Cerrobend inserts passed a 2%/1 mm criteria for 100% of the area.

The use of milled copper inserts resulted in lower out‐of‐field dose compared to Cerrobend inserts. This difference increased at higher energies and with larger applicators, and decreased at extended SSD (110 cm). This effect caused the 2%/1 mm criteria failures for the small field size‐large applicator combinations at the highest measured energy (20 MeV). Clinically, this dosimetric difference could be beneficial in treatment as the use of copper inserts can reduce the dose received by healthy tissue outside of the planned treatment volume and could also allow for more homogenous dose distributions during field abutment.

The use of milled copper inserts resulted in a slightly higher in‐field dose near the beam edge compared to Cerrobend inserts at shallow depths (<2cm). However, this effect had no significant effect on the absolute dose comparisons (<2%). For all field size‐applicator‐energy combinations at 100 cm and 110 cm SSD, dosimetric comparisons showed 100% of the area passing the 2%/1 mm criteria inside the area of clinical beam.

The output from Cerrobend inserts can be as much as 1.7% lower or 0.9% higher than for copper inserts (Table 4). However, these differences occur for unlikely applicator–field size combinations (i.e., small fields with a large applicator). It is recommended that, when planning with copper inserts, the institution decide on the acceptability of this difference. The data found in Rusk ^(6)^ can be used to determine the appropriate correction factors, if necessary.

Therefore, it should be clinically acceptable to utilize copper inserts with dose distributions and dose outputs measured with Cerrobend inserts for treatment planning dose calculations and monitor unit calculations.

## COPYRIGHT

This work is licensed under a Creative Commons Attribution 3.0 Unported License.

## Supporting information

Supplementary Material
