# Supplementary material for: A dosimetric comparison of copper and Cerrobend electron inserts
Source: J Appl Clin Med Phys. 2016 Sep 8;17(5):245–61. doi: 10.1120/jacmp.v17i5.6282 (PMC5874111; doi:10.1120/jacmp.v17i5.6282)
Supplement: Supplementary file 1 — Supplementary Material [file ACM2-17-245-s001.docx]

A Dosimetric Comparison of Copper and Cerrobend Electron Inserts

**Abstract**: The purpose of this work was to evaluate differences in dose resulting from use of copper aperture inserts compared to lead-alloy (Cerrobend®) aperture inserts for electron beam therapy. Specifically, this study examines if copper aperture inserts can be used clinically with the same commissioning data measured using lead-alloy aperture inserts.

The copper inserts were acquired from .decimal, Inc. and matching lead-alloy, Cerrobend®, inserts were constructed in-house for 32 combinations of nine square field sizes (2x2 to 20x20 cm^2^) and five applicator sizes (6x6 to 25x25 cm^2^). Percent depth-dose and off-axis relative dose profiles were measured using an electron diode in water for the copper and Cerrobend® inserts for a subset of insert sizes (6x6, 10x10, 25x25 cm^2^) and energies (6, 12, 20 MeV) at 100 and 110 cm source-to-surface distances (SSD) on a Varian Clinac 21EX accelerator. Dose outputs were measured for all field size-insert combinations and five available energies (6-20 MeV) at 100 cm SSD and for a smaller subset at 110 cm SSD. Using these data, 2D planar absolute dose distributions were generated and compared. Criteria for agreement were ±2% of maximum dose or 1 mm distance-to-agreement for 99% of points.

A gamma analysis of the beam dosimetry showed 94 of 96 combinations of insert size, applicator, energy, and SSD were within the 2%/1 mm criteria for > 99% of points. Out-of-field, copper inserts showed less bremsstrahlung dose under the insert compared to Cerrobend® (greatest difference was 2.5% at 20 MeV and 100 cm SSD). This effect was most prominent at the highest energies for combinations of large applicators with small field sizes, causing some gamma analysis failures. Inside the field, more electrons scattered from the collimator edge of copper compared to Cerrobend®, resulting in an increased dose at the field edge for copper at shallow depths (greatest increase was 1% at 20 MeV and 100 cm SSD). Dose differences decreased at the SSD increased, with no gamma failures at 110 cm SSD. Inserts for field sizes ≥6x6 cm^2^ at any energy, or for small fields (≤4x4 cm^2^) at energies <20 MeV, showed dosimetric differences less than 2%/1 mm for more than 99% of points. All areas of comparison criteria failures were from lower out-of-field dose under copper inserts due to a reduction in bremsstrahlung production, which is clinically beneficial in reducing dose to healthy tissue outside of the planned treatment volume. All field size-applicator size-energy combinations passed 3%/1 mm criteria for 100% of points.

Therefore, it should be clinically acceptable to utilize copper insets with dose distributions measured with Cerrobend inserts for treatment planning dose calculations and monitor unit calculations.

**I. Introduction**

Electron therapy treatments typically use patient-specific devices to conform the lateral beam edges to the physician outlined planning treatment volume (PTV). The most common device used is the electron applicator, which attaches below the treatment head and includes three trimmers to further collimate the beam along with a bottom tray for patient-specific collimation. While photon treatments can use multileaf collimators (MLCs) to achieve patient-specific collimation, electron MLC prototypes exist but are not commonly available in commercial treatment machines.^1, 2^ Instead, electron beams use custom inserts for shaping of the lateral field edges to conform to the PTV while sparing adjacent critical structures and normal tissue.

The most commonly used electron insert, introduced by Powers et al., is Lipowitz metal, known by the brand name Cerrobend®.^3^ To create a custom insert the field shape is traced onto and then cut out of a block of Styrofoam, usually with a heated wire. The Styrofoam cutout is placed in a mold which is then filled with molten Cerrobend. This technique for creating custom inserts for external beam therapy has remained relatively unchanged since the 1970’s, however it does hold some disadvantages such as laborious fabrication with challenging reproducibility, as well as environmental and safety precautions.^4^

Custom insert creation requires a separate room for the applicator molds, melting vat, Cerrobend inventory, and safety equipment. These block cutting rooms are primarily used for electron inserts, which typically make up a small percentage (<15%) of total treatments. Many centers only treat with custom electron inserts a few times a year, making the maintenance of a block cutting room highly inefficient. In-house insert creation also suffers from inaccuracies due to manually cutting Styrofoam and pouring Cerrobend. The effects of those inaccuracies are magnified when there is a need for high precision, such as cases with adjacent critical structures or abutting fields. Additionally, staff therapists or dosimetrists must be trained in insert creation techniques and proper safety when handling Cerrobend because of the presence of toxic lead and cadmium. Employing an outside vender to mill the patient-specific inserts makes it possible to eliminate block cutting rooms, and use alternative materials, such as copper, an environmentally friendly, non-toxic and easily machinable material

Copper is more robust than Cerrobend, making it less likely to be damaged from repeated use or accidental drops. Third party vendors such as .decimal Inc. (Sanford, FL) machine custom inserts from patients’ treatment planning files, with devices received by the treatment center within 1-3 days of request. (<http://dotdecimal.com/products/electrons/apertures/>).

In addition, small centers lacking the equipment or training required to create their own inserts can still treat patients with electron beam therapy using vendor-supplied inserts. Shipping time and cost are two disadvantages of using third party vendors. Also, small modifications of the aperture during clinical setup are more difficult.

Of clinical concern when using copper inserts is the validity of Cerrobend-based commissioning data used for dose and monitor unit (MU) calculations in treatment planning and MU calculation systems. Many linear accelerators were commissioned using Cerrobend inserts to determine percent depth dose curves (PPD), off-axis relative dose profiles, output factors, and air gap factors. Differences in these dosimetric data between Cerrobend and copper inserts can decrease the accuracy of the treatment planning system’s electron dose calculations. Prior to implementing copper inserts for use in patient treatment, it is necessary to investigate dosimetric differences between copper and Cerrobend electron inserts.

Standard commissioning beam metrics (PDDs, off-axis relative dose profiles at various depths, and output factors) were measured for a clinically relevant range of applicator sizes (6x6-25x25 cm^2^), insert sizes (2x2-20x20 cm^2^), energies (6-20 MeV), and SSDs (100 and 110 cm) for the Varian Clinac 21EX at Mary Bird Perkins Cancer Center (Baton Rouge, LA). These beam metrics were combined to generate absolute 2D dose distributions, which were evaluated for clinically significant differences caused by the use of copper inserts compared to Cerrobend inserts.

**II. Methods**

All electron beam dosimetric data were measured at MBPCC on a Varian Clinac 21EX 4/10 linear accelerator (SN: 1412), following the guidelines from TG-106.^4^ Electron energies available were 6, 9, 12, 16, and 20 MeV (E_p,0_ = 5.95, 8.76, 12.51, 16.36, and 19.68 MeV). Applicators used were Varian Type III accessories in sizes of 6x6, 10x10, 15x15, 20x20, and 25x25 cm^2^. All measurements (PDDs, off-axis profiles, and output factors) were relative dose measurements.

**II.A. Creation of a Matching Set of Copper and Cerrobend Inserts**

**II.A.1. Creating the Matching Set of Inserts**

To compare dosimetric differences between copper and Cerrobend inserts for potential clinical use, dose distributions were measured spanning the clinically relevant range of applicators (6x6 cm^2^-25x25 cm^2^) and field sizes (2x2 cm^2^-20x20 cm^2^ defined at isocenter). Table I shows 32 field size-applicator combinations studied. No dose distribution measurements were taken for the open field sizes, because these field size-applicator combinations do not require custom inserts.

Inserts were constructed to be of sufficient thickness for the highest energy (20 MeV), a standard procedure for clinical practice, which allows the same insert to be used at all energies (6-20 MeV). The required minimum thickness of lead (t_Pb_) for shielding a 20 MeV electron beam, maximum energy of electron beams ranging from 6-20 MeV, according to AAPM task group 25 is 10 mm.^5^ Density scaling from lead (ρ_Pb_=11.34 g/cm^3^) to Cerrobend (ρ_Cerrobend_=9.38 g/cm^3^) and copper (ρ_Cu_=8.96 g/cm^3^) was used to calculate the minimum required thicknesses at 20 MeV of 11.9 cm and 12.5 cm, respectively. Note that the copper inserts must be ~5.5% thicker than Cerrobend to achieve the same electron shielding.

The 32 copper inserts were milled by .decimal Inc. along with corresponding aluminum negatives. These negatives were used to mold a matching set of Cerrobend inserts at Mary Bird Perkins Cancer Center (MBPCC). Figure 1 shows Cerrobend poured into a 15x15 cm^2^ applicator mold tray around a centered aluminum negative for a 4x4 cm^2^ field, and the resulting Cerrobend insert alongside its matching copper insert.

### II.A.2 Quality Assurance of Matching Electron Inserts

Thickness measurements were taken using a digital caliper with a 0.002 cm precision. In addition, the square field size in the X and Y direction of each insert was measured using a digital caliper with 0.002 cm precision, except for the largest field size (20x20 cm^2^), which was measured using a ruler with 0.5 mm precision.

The average measured thickness of all the copper inserts was 14.80 mm with a standard deviation of 0.07 mm. The average measured thickness of all Cerrobend inserts was 12.46 mm with a standard deviation of 0.89 mm. The electron transmitted dose (D-D_bremsstrahlung_) is less than 2% of maximum dose with no shielding in this energy range, with residual levels being due to bremsstrahlung produced photons, rather than electron transmission. Therefore differences in electron transmission through the copper and Cerrobend inserts is expected to be negligible. A complete table of all copper and Cerrobend insert thicknesses can be found in Rusk’s master’s thesis.^6^

The average difference between the X and Y field size for every insert was 0.07 mm with a standard deviation of 0.08 mm for copper and 0.15 mm with a standard deviation of 0.13 mm for Cerrobend. The mean of the X and Y measurements for each insert formed the average field size. Comparing the average field sizes between matching copper and Cerrobend inserts, the average difference (Cerrobend minus copper) in field sizes was 0.15 mm with a standard deviation of 0.10 mm and a maximum difference of 0.33 mm. These differences are negligible (<0.5 mm), so the copper and Cerrobend inserts were treated as having the same field sizes.

## II.B. Measurement of Dosimetric Data

**II.B.1 Measurement Equipment**

Dose measurements were made using a p-type electron dosimetry diode detector (IBA EFD^3G^, #300-605) with an active volume diameter of 2 mm and thickness of 0.06 mm. Silicon-diode detectors measure ionization in the active region of the diode, where it is assumed that ionization is proportional to dose, i.e. correction factors are energy independent. These detectors can be used to accurately measure relative dose distributions for high energy electron beams.^7, 8^

The diode was connected to the beam scanning main control unit (MCU), which contained an internal electrometer for PDD and off-axis relative dose profile measurements. For output measurements the diode was connected to an external calibrated electrometer. This arrangement allowed the use of the 2D scanning motors to precisely position the diode at depths for output readings using the MCU software. Since the diode and water phantom setup remained unchanged between scans and output measurements, the setup also ensured consistency in the geometry for all measurements.

All PDDs, off-axis relative dose profiles, and output measurements (100 cm SSD) were taken in a RFA-200 Water Phantom 2D scanning tank using OmniPro scanning software (IBA Dosimetry). The phantom was leveled in all directions to assure scanning would be aligned with the electron beam. The diode was placed near the center of the phantom and the couch adjusted laterally and longitudinally to align the diode with the center of the light field from the linear accelerator. The couch was adjusted vertically to the desired SSD using mechanical distance indicators. Periodically the SSD was verified by using the optical distance indicator (ODI). The flat entry surface of the diode was then visually set even to the water surface and its position was zeroed in the scanning software, by adjusting for the known effective measurement location. Because of the long duration of scanning, care was taken to maintain a constant water level. The water level was checked regularly throughout the day and water added to compensate for any evaporation.

Quality assurance of the measuring apparatus was done daily to ensure the mechanical stability of the scanning equipment as well as energy stability of the linear accelerator. QA for the mechanical scanning equipment was performed by taking three consecutive PDD scans of the 9 MeV beam at least once per day. Verifying that the R_50_ values of the three scans were within a tolerance of ±0.05 cm ensured that the mechanical components of the 2D scanning phantom were operating properly.

QA for energy stability was performed by measuring a 9 MeV PDD using the open field size in whichever applicator was being used for the measurements that day. The beginning-of-day and end-of-day PDDs were then compared to ensure that the R_50_ values were within a tolerance of ±0.1 cm. This ensured that the energy of the 9 MeV beam had not changed throughout the day, that the beam scanner was aligned properly, and that the diode and electrometer were functioning properly. The 9 MeV beam was chosen because of its sharp falloff in the PDD, which facilitates R_50_ measurement while also having greater depth of penetration than the 6 MeV beam.

### II.B.2 Measurement Subsets

Measurement subsets were chosen to span the range of clinical combinations of energy, applicator, field size, and SSD. PDD curves and outputs at 100 cm SSD were measured for all five available energies (6, 9, 12, 16, and 20 MeV) for all field size-applicator combinations (Table I). Off-axis relative dose profiles were measured for copper and Cerrobend inserts using three energies (6, 12, and 20 MeV) at 100 cm and 110 cm SSD for the field size-applicator combinations shown in Table II. This subset was also used for measuring PDD curves and outputs at 110 cm SSD. This subset was chosen to sample field sizes using the smallest, middle, and largest sized applicators available on the Varian machine. Most clinical electron beam treatments use an energy, SSD, and field size/applicator size geometry in the range spanned by this subset.

### II.B.3. Percent Depth Dose Curves

PDD curves were measured using the OmniPro® scanning software with a 1 mm step size and low scan speed in precision mode. All beam scans followed the guidelines described by TG-25^5, 9^ and TG-51.^10^ PDD scans were made from deeper to shallower depths, beginning at depths of 8, 12, and 14 cm for energies of 6, 12, and 20 MeV respectively. These PDDs were used to compare beam metrics and to create isodose plots for evaluation and comparison. In addition, a more extensive subset was used for measuring PDD curves and outputs at 100 cm SSD. These measurements were taken at all five available energies (6, 9, 12, 16, and 20 MeV) for all field size-applicator combinations.

### II.B.4. Off-Axis Relative Dose Profiles

Off-axis relative dose profiles were measured with the OmniPro® scanning software using a step size of 2 mm and a low scan speed in precision mode. The off-axis relative dose profiles were measured immediately after the PDDs for each insert. A scan consisted of off-axis profiles measured at a number of depths beginning 0.5 cm below the surface of the water. The number and depths of the off-axis relative dose profiles was selected for each energy to acquire data in the high gradient regions and to cover the entire practical range of the beam. Eleven off-axis profiles were measured for 6 MeV beams (depths of 0.5, 1, 1.5, 2, 2.25, 2.5, 2.75, 3, 3.5, 4, and 5 cm), 15 profiles for 12 MeV beams (depths of 0.5, 1, 1.5, 2, 2.5, 3, 3.5, 4, 4.5, 5, 5.5, 6, 6.5, 7, and 8 cm), and 22 profiles for 20 MeV beams (depths of 0.5, 1, 1.5, 2, 2.5, 3, 3.5, 4, 4.5, 5, 5.5, 6, 6.5, 7, 7.5, 8, 8.5, 9, 9.5, 10, 11, and 12 cm). Margins of 4 cm were added to the diverging field edge projecting to the deepest profile, and that width was used for all profile measurements. These measurements allowed significant data for evaluation of out-of-field dosimetry. The off-axis data were combined with PDDs to construct a full 2D dose grid from which isodose curves could be plotted.

### II.B.5. Output Correction Factors

The output correction factor (OCF) is defined (eqn. 1) as the ratio of the average copper output reading at the R_100_ for Copper divided by the average Cerrobend output reading at the R_100_ for Cerrobend for a particular energy, applicator, field size, and SSD. The ratio of outputs equals the ratio of charge outputs for these measurements. Electron beam relative outputs were measured at 100 cm SSD using a 2D water phantom and at 110 cm SSD using a 1D water phantom (Standard Imaging, DoseView 1D). Relative dose measurements were taken at R_100_ using an external electrometer (CNMC Model 206 dosimetry electrometer) and the same electron diode used for the PDDs and off-axis relative dose profile measurements. The internal electrometer of the IBA MCU was not designed for measuring the output of a single diode. Therefore, a cable connecting the diode to the external electrometer outside of the vault allowed for easy transition from the MCU to an external electrometer.

The electron inserts were aligned with the central axis using the etchings for copper inserts and a ruler for the Cerrobend inserts. The diode was centered using the linear accelerator’s crosshairs. Initial off-axis profile scans were taken with each new insert to check this centering. Each centering profile was taken in-plane at a depth of 1 cm. Inserts were considered properly centered if the measured off-axis profile centers were <0.05 cm from the beam center, with couch adjustments used to align the diode with the beam center as necessary. These measurements ensured that the diode was aligned with the radiation central axis.

After ensuring the diode was centered, a PDD was measured to determine R_100_. Using OmniPro®, the diode was repositioned to R_100_. The diode detector was then disconnected from the MCU and connected to the external electrometer. Three electrometer readings were recorded, each with the machine delivering 200 monitor units (MUs) and then averaged. Cerrobend and copper insert outputs were measured at the R_100_ corresponding to Cerrobend. This process was repeated for all five beam energies for a single Cerrobend insert, and then repeated for the matching copper insert immediately afterwards. Consecutively measuring the matching Cerrobend and copper inserts resulted in less than 30 minutes between measurement sets of the same energy and insert size for the two materials.

There was close agreement between R_100_ values for Cerrobend and copper inserts, with an average difference of less than 0.1 cm. The differences in R_100_ locations all resulted in %DD corrections of less than 0.1%. As such, the OCFs were calculated without %DD corrections, as shown in equation 1. The uncertainty for the OCF calculations was estimated to be ±0.001.^6^

|  | $OCF\left( E,Appl,FS,SSD \right)=\frac{O^{Cu}\left( E,Appl,FS,SSD \right)}{O^{Cerrobend}\left( E,Appl,FS,SSD \right)}$ | (1) |
| --- | --- | --- |

The OCFs were computed for both copper and Cerrobend at 100 and 110 cm SSD for the measurement subsets shown in Tables I and II, respectively.

### II.C. Comparison of Beam Dosimetry

### II.C.1. Data Processing

Prior to the comparison of absolute beam dosimetry, post processing of the raw data in Omni pro was done in the following order. (1) PDD data were normalized to 100% at the depth of maximum dose (R­_100_); (2) off-axis relative dose profiles were centered; (3) off-axis profiles were symmetrized using the mean value from both sides; and (4) profiles were renormalized to the central axis value (from the PDD). No smoothing filters were applied to any scan.

**II.C.2. Creation of Absolute 2D Dose Distributions**

The OCFs were used to scale the relative dose distributions for copper inserts to create “absolute” dose distributions. Absolute 2D dose distributions are the relative dose distributions normalized such that 100% corresponds to the central-axis dose measurement at R_100_ for Cerrobend (Equations 2 and 3), i.e.

|  | $D_{Absolute}^{Copper}(x,z)=D_{Relative}^{Copper}(x,z)\times OCF$ | (2) |
| --- | --- | --- |
|  | $D_{Absolute}^{Cerrobend}(x,z)=D_{Relative}^{Cerrobend}(x,z)$ | (3) |

Absolute 2D dose distributions for matching copper and Cerrobend inserts under the same measurement conditions (i.e. applicator, field size, energy, SSD) were overlaid with isodose lines plotted for visual interpretation of the dose distributions.

### II.C.3 Comparison Criteria

To implement copper inserts clinically without re-commissioning, i.e. using dosimetry data previously measured with Cerrobend inserts, the dosimetric differences between copper and Cerrobend inserts must be clinically acceptable. Annual quality assurance procedures from TG-40^11^ and TG-142^12^ recommend measuring a subset of the commissioning data and comparing to the baseline data to determine dosimetric accuracy, as was done in this study. The dosimetric tolerances described by these Task Group reports were used as a comparison criteria.

Cerrobend and copper insert dosimetry data were compared quantitatively on dose distributions with the same delivery geometry. Using a 2%/1 mm criteria, the superimposed dose distributions were checked at each point for agreement to within 2% of the central axis maximum dose for Cerrobend, i.e. the 100% point, or a point which agrees within a radius of 1 mm in the dose measurement plane. The percentage of points passing the criteria was recorded. Any comparison containing failing points were re-analyzed using a 3%/1 mm criteria.

The dosimetric criteria of ±2% of maximum dose or ±1 mm distance to agreement (DTA), consistent with TG-142, was used as a metric for output factors and beam quality. Analysis was performed using the percent of maximum dose difference rather than simply the percent difference because of the greater clinical significance of percent of maximum dose.

**III. Results and Discussion**

## III.A. Measurement of Dosimetric Data

### III.A.1 Percent Depth Dose Curves at 100 cm SSD

Percent depth dose (PDD) curves at 100 cm SSD were measured for all field size (2x2-20x20 cm^2^) and applicator (6x6-25x25 cm^2^) combinations shown in Table I for all energies 6-20 MeV using both Cerrobend and copper inserts. Measured percent depth dose curve comparisons between copper and Cerrobend at 100 cm SSD are shown for all energies and a sampling of field sizes (2x2 – 20x20 cm^2^) in the 25x25 cm^2^ applicator (Figure 2). The most notable differences were at deeper depths for the smaller field sizes (2x2 and 4x4 cm^2^), possibly due to decreased bremsstrahlung production in the copper insert. Overall, PDDs showed negligible (<1%/1 mm) differences between copper and Cerrobend for the entire PDD curves (surface, peak and fall-off regions).

Percent depth dose metrics were compared at 100 cm SSD for the 160 pairs of PDDs arising from the 32 field size and applicator size combinations, five energies, and two materials. Metrics included R_50_, R_90_, and R_80-20_. The dose at 1.0 cm depth (D_1.0_) was also compared between the inserts to examine dose differences at shallow depths. The differences in each of these metrics between matching copper and Cerrobend inserts at the same energy were calculated by taking the Cerrobend value minus the copper value.

All PDD metric comparisons averaged over all field sizes showed negligible differences (<0.1 cm) between copper and Cerrobend inserts. The maximum differences for R_50_, R_90_, and R_80-20_ were 0.07 cm, 0.13 cm, and 0.08 cm, respectively. The maximum difference in D_1.0_ between the two materials was -0.90% of central axis dose maximum. All 100 cm PDD plots and metric data can be found in Appendix A of Rusk.^6^

### III.A.2 Percent Depth Dose Curves at 110 cm SSD

Percent depth dose curves at 110 cm SSD were measured for the subset of field size-applicator combinations listed in Table II for energies of 6 MeV, 12 MeV, and 20 MeV. The PDDs showed negligible (<1%/1 mm) differences between copper and Cerrobend for the entire PDD curves (surface, peak and fall-off regions). Measured PDD comparisons between copper and Cerrobend at 110 cm SSD are shown in Figure 3 for all energies and a series of field sizes in the 25x25 cm^2^ applicator.

The same percent depth dose metrics were compared at 110 cm SSD as 100 cm SSD for the 48 pairs of PDDs arising from 16 field size/applicator size combinations, 3 energies, and 2 materials. All PDD metric comparisons showed negligible differences between copper and Cerrobend inserts. The maximum differences for R_50_, R_90_, and R_80-20_ were 0.01 cm, 0.02 cm, and 0.09 cm, respectively. The maximum difference in D_1.0_ between the two materials was 0.80% of central axis dose maximum. All 110cm PDD plots and metric data can be found in Appendix B of Rusk.^6^

### III.A.3. Central Axis Photon Dose

Percent depth dose curves showed little variation between copper and Cerrobend at depths smaller than the practical range (R_P_) for all energies and field size combinations. However, the bremsstrahlung dose (D_x_) in the tail region of the PDDs did show consistent differences (≥0.1%) at energies 12 MeV and higher. As energy and applicator size increased, and as field size decreased the difference in D_x_ (Cerrobend minus copper) increased, as shown in Table III.

Bremsstrahlung production increased with the surface area of the insert material in the beam. A 2x2 cm^2^ field size in a 25x25 cm^2^ applicator has 19.4 times more insert material being struck by the electron beam than a 2x2 cm^2^ field size in a 6x6 cm^2^ applicator. This increased bremsstrahlung production to the center of the field can result in up to a 0.5% of maximum dose increase in central axis dose for Cerrobend inserts compared to copper inserts This effect is greatest when the field is small (2x2 cm^2^), the applicator is large (25x25 cm^2^), and high energy (20 MeV).

### III.A.4. Off-Axis Relative Dose Profiles

Measured off-axis relative dose profiles from copper and Cerrobend inserts had good overall agreement for all energies, SSDs, and insert/applicator combinations with differences predominantly <2% of maximum dose. Off-axis relative dose profiles showed the greatest differences between copper and Cerrobend at the most shallow depth of 0.5 cm. The observed dosimetric differences were located near the beam edge inside the field and in out-of-field regions, as shown in Figure 4. The maximum in field differences for the 6, 12, and 20 MeV energies are 0.5%, 0.7%, and 1.0%, respectively. The maximum out-of-field differences for the 6, 12, and 20 MeV energies are 0.5%, 0.7%, and 1.0%, respectively.

Off-axis relative dose profiles showed higher doses inside the beam edges for copper inserts than for Cerrobend inserts at depths of less than ~2 cm, being more prominent with higher energy beams and shallower depths. At depths past ~2cm no differences in the dose inside the beam edges between the two materials were observed. These beam-edge horns were attributed to electrons scattering from the collimator edge, illustrated by an inverse relationship between the absorbed dose from scatter and the density of the collimating material.^13, 14^ The lower density of copper compared to Cerrobend caused more electron scatter from the insert edge, and thus a higher dose at the field edge. These edge effects never exceeded 2% of central-axis maximum dose.

Dosimetric differences were also seen in the out-of-field region shielded by the insert at off-axis distances greater than ~2 cm outside the beam edge. While the 6 MeV energy showed no distinct differences in out-of-field dose, off-axis profiles showed higher out-of-field doses for Cerrobend than copper at 12 MeV and 20 MeV, with these differences most noticeable at 20 MeV where they sometimes exceeded the ±2% tolerance. As the depth of the measured profile increased this difference became less pronounced.

This higher out-of-field dose from Cerrobend inserts as compared to copper inserts was attributed to the relative decrease in the amount of bremsstrahlung production in copper, discussed previously. Out-of-field doses from Cerrobend showed dose increases >2% of maximum dose compared to copper for some inserts. This effect gives copper inserts a clinical advantage in lowering the risk of secondary cancers by reducing the out-of-field dose.

### III.A.5 Output Correction Factors

OCFs (copper/Cerrobend) at 100 cm SSD ranged from 0.983 (3x3 cm^2^ field size/25x25 cm^2^ applicator/16 MeV) to 1.009 (6x6 cm^2^ field size/10x10 cm^2^ applicator/20 MeV). All OCFs at 100 cm SSD were within ±2% of unity. The average OCF was 0.999, with Cerrobend having a 0.1% average higher output than copper, as shown in Table IV. Table V shows a subset of OCFs covering the clinical range of energies, field sizes, and applicators. Measured average OCFs were 0.999 at 6 MeV, 9 MeV, and 12 MeV, while the average OCF was 0.998 at 16 MeV and 20 MeV. The slightly higher average outputs from Cerrobend inserts compared to copper inserts could be caused by the greater bremsstrahlung production in Cerrobend, especially at higher energies. A table of all OCF for 100 SSD cm can be found in Appendix C of Rusk.^6^

OCFs at 110 cm SSD ranged from 0.990 (2x2 cm^2^ field size/25x25 cm^2^ applicator/6 MeV, 3x3 cm^2^ field size/25x25 cm^2^ applicator/20 MeV, and 4x4 cm^2^ field size/25x25 cm^2^ applicator/20 MeV) to 1.006 (4x4 cm^2^ field size/15x15 cm^2^ applicator/6 MeV and 8x8 cm^2^ field size/15x15 cm^2^ applicator/6 MeV). All OCFs at 110 cm SSD were within ±1% of unity. For 110 cm SSD, the average OCF over all energies was 0.999, with 6 MeV having the largest average (1.001) and 20 MeV the smallest average (0.997), as shown in Table VI. Table VII shows a subset of OCFs covering the clinical range of energies, field sizes, and applicators. The slightly higher average outputs from Cerrobend inserts compared to copper inserts at 20 MeV might be caused by the greater bremsstrahlung production in Cerrobend at higher energies. A table of all OCF for 110 SSD cm can be found in Appendix D of Rusk.^6^

## III.B. Comparison of Beam Dosimetry

### III.B.1. Analysis of Isodose Plots at 100 cm SSD

Figure 5 shows isodose comparisons between copper and Cerrobend dose distributions for the 12x12 cm^2^ field in the 15x15 cm^2^ applicator for the 6, 12, and 20 MeV beams. Figure 6 shows isodose comparisons between copper and Cerrobend dose distributions for the 12x12 cm^2^ field in the 15x15 cm^2^ applicator for the 6, 12, and 20 MeV beams. Isodose plots for all measured dose distribution comparisons at 100 cm SSD can be found in Appendix E of Rusk.^6^

Of the 48 total combinations of field size, applicator size, and energy, 43 (90%) passed the 2%/1 mm criteria for 100% of points. For 46 of 48 (96%) combinations, ≥99% of points passed the 2%/1 mm criteria; the two failing combinations were the 2x2 cm^2^ field size (98.90% passing) and the 4x4 cm^2^ field size (98.35% passing) both in the 25x25 cm^2^ applicator at 20 MeV. The other three combinations showing point failures were the next three largest field sizes in the 25x25 cm^2^ applicator at 20 MeV: 6x6 cm^2^ field size (99.44% passing), 8x8 cm^2^ field size (99.92% passing) and the 10x10 cm^2^ field size (99.67% passing).

At 20 MeV, the additional out-of-field bremsstrahlung dose produced in the Cerrobend inserts compared to the copper inserts caused the observed failures in the 2%/1 mm criteria. The off-axis dose due to bremsstrahlung photons is higher for the Cerrobend insert than the copper insert by a maximum of 2.2% at 0.5 cm depth in this off-axis region. A detailed analysis of the off-axis bremsstrahlung differences can be found in Rusk.^6^

All criteria failures occurred out-of-field due to the lower bremsstrahlung dose from copper inserts compared to Cerrobend inserts, a difference which reduces dose to healthy tissue and is clinically beneficial. Clinically, an insert is created using the smallest possible applicator for the given field size. The inserts which registered criteria failures in this study were small field size-large applicator combinations which are unlikely to be used for patient treatment.

The increased scatter through the edge of copper inserts compared to Cerrobend inserts did not result in criteria failure for any of the isodose comparisons. While this increased scatter was noticeable in some off-axis relative dose profiles at shallow depth, the increased scatter did not impact the isodose comparisons. Maximum differences in the beam edge region for copper inserts was less than the 2%/1 mm criteria when compared to Cerrobend inserts.

**III.B.2 Analysis of Isodose Plots at 110 cm SSD**

Isodose comparisons between the copper and Cerrobend inserts using the 2%/1 mm DTA criteria at 110 cm SSD showed all 48 of 48 comparisons passing the criteria for 100% of points. Figures 7 and 8 show representative samples of the best and worst agreement, respectively, between the Cerrobend and copper dose distributions. The increased out-of-field dose from the Cerrobend inserts at extended SSD as compared to the copper inserts was still apparent. However, the differences between the out-of-field doses were less than those observed at 100 cm SSD. Isodose plots for all measured dose distribution comparisons at 110 cm SSD can be found in Appendix F of Rusk.^6^

**IV. Summary and Recommendations**

Using custom milled copper inserts for electron beam therapy planned with standard commissioning data measured on Cerrobend inserts should result in minimal absolute dosimetric differences (≥99% of points within ±2% of D_max_ or 1 mm DTA) for standard clinical field sizes (2x2 cm^2^ to 20x20 cm^2^), applicators (6x6 cm^2^ to 25x25 cm^2^), and energies (6 MeV to 20 MeV). At 100 cm SSD, all dosimetric comparisons of copper and Cerrobend inserts passed a 3%/1 mm criteria for 100% of the area. At 100 cm SSD, comparisons of the absolute dosimetric difference between copper and Cerrobend inserts showed 100% of the area within 2%/1 mm agreement for all field sizes at energies of 6 and 12 MeV. Only copper and Cerrobend small field inserts (2x2 cm^2^ and 4x4 cm^2^) in the 25x25 cm^2^ applicator at 20 MeV resulted in less than 99% of the area passing the 2%/1 mm comparison criteria, with the worst case being a 98.35% passing rate for the 4x4 cm^2^ field in the 25x25 cm^2^ applicator. At 110 cm SSD, all dosimetric comparisons of copper and Cerrobend inserts passed a 2%/1 mm criteria for 100% of the area.

The use of milled copper inserts resulted in lower out-of-field dose compared to Cerrobend inserts. This difference increased at higher energies and with larger applicators, and decreased at extended SSD (110 cm). This effect caused the 2%/1 mm criteria failures for the small field size-large applicator combinations at the highest measured energy (20 MeV). Clinically, this dosimetric difference could be beneficial in treatment as the use of copper inserts can reduce the dose received by healthy tissue outside of the planned treatment volume and could also allow for more homogenous dose distributions during field abutment.

The use of milled copper inserts resulted in a slightly higher in-field dose near the beam edge compared to Cerrobend inserts at shallow depths (<2 cm). However, this effect had no significant effect on the absolute dose comparisons (<2%). For all field size-applicator-energy combinations at 100 cm and 110 cm SSD, dosimetric comparisons showed 100% of the area passing the 2%/1 mm criteria inside the area of clinical beam.

Therefore, it should be clinically acceptable to utilize copper insets with dose distributions measured with Cerrobend inserts for treatment planning dose calculations and monitor unit calculations.

#### V. References

^1^Lee M C, Jiang S B and Ma C M, “Monte Carlo and experimental investigations of multileaf collimated electron beams for modulated electron radiation therapy”, *Med Phys* **27** 2708-18 (2000).

^2^Hogstrom K R, Boyd R A, Antolak J A, Svatos M M, Faddegon B A and Rosenman J G, “Dosimetry of a prototype retractable eMLC for fixed-beam electron therapy”, *Med Phys* **31** 443-62 (2004).

^3^Powers W E, Kinzie J J, Demidecki A J, Bradfield J S and Feldman A,”A new system of field shaping for external-beam radiation therapy”, *Radiology* **108** 407-11 (1973).

^4^Das I J, Cheng C-W, Watts R J, Ahnesjö A, Gibbons J, Li X A, Lowenstein J, Mitra R K, Simon W E and Zhu T C ,“Accelerator beam data commissioning equipment and procedures: Report of the TG-106 of the Therapy Physics Committee of the AAPM”, *Med Phys* **35** 4186 (2008).

^5^Khan F M, Doppke K P, Hogstrom K R, Kutcher G J, Nath R, Prasad S C, Purdy J A, Rozenfeld M and Werner B L, “Clinical electron-beam dosimetry: report of AAPM Radiation Therapy Committee Task Group No. 25”, *Med Phys* **18** 73-109 (1991).

^6^Rusk, Benjamin D., “A dosimetric comparison of copper and Cerrobend electron insets”, Masters Thesis. (Baton Rouge, Louisiana: Physics and Astronomy. Louisiana State University) (<http://etd.lsu.edu/docs/available/etd-06162014-153414/>).

^7^Rikner G 1985 Characteristics of a p-Si detector in high energy electron fields *Acta radiologica. Oncology* **24** 71-4

^8^Ten Haken R K, Fraass B A and Jost R J, “Practical methods of electron depth-dose measurement compared to use of the NACP design chamber in water”, *Med Phys* **14**, 1060-6 (1987).

^9^Gerbi B J, Antolak J A, Deibel F C, Followill D S, Herman M G, Higgins P D, Huq M S, Mihailidis D N, Yorke E D, Hogstrom K R and Khan F M 2009 Recommendations for clinical electron beam dosimetry: Supplement to the recommendations of Task Group 25 *Medical Physics* **36** 3239

^10^Almond P R, Biggs P J, Coursey B M, Hanson W F, Huq M S, Nath R and Rogers D W 1999 AAPM's TG-51 protocol for clinical reference dosimetry of high-energy photon and electron beams *Med Phys* **26** 1847-70

^11^Kutcher G J, Coia L, Gillin M, Hanson W F, Leibel S, Morton R J, Palta J R, Purdy J A, Reinstein L E, Svensson G K and et al. 1994 Comprehensive QA for radiation oncology: report of AAPM Radiation Therapy Committee Task Group 40 *Med Phys* **21** 581-618

^12^Klein E E, Hanley J, Bayouth J, Yin F F, Simon W, Dresser S, Serago C, Aguirre F, Ma L, Arjomandy B, Liu C, Sandin C, Holmes T and Task Group A A o P i M 2009 Task Group 142 report: quality assurance of medical accelerators *Med Phys* **36** 4197-212

^13^Lax I and Brahme A 1980 Collimation of high energy electron beams *Acta radiologica. Oncology* **19** 199-207

^14^International Commission on Radiation Units and Measurements. 1972 *Radiation dosimetry: electrons with initial energies between 1 and 50 MeV* (Washington,

**VI. Figures**


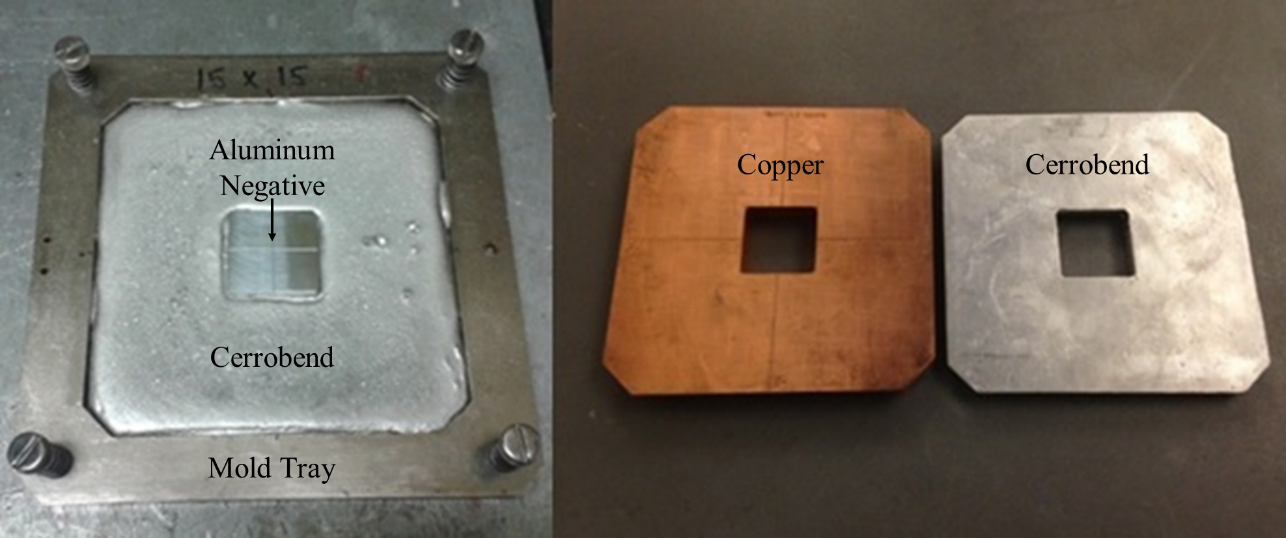


Figure 1: (LEFT) Photo of Cerrobend poured into a 15x15 cm^2^ applicator mold

to generate an insert formed by a 4x4 cm^2^ aluminum negative. (RIGHT) The resulting Cerrobend insert alongside its matching copper insert.


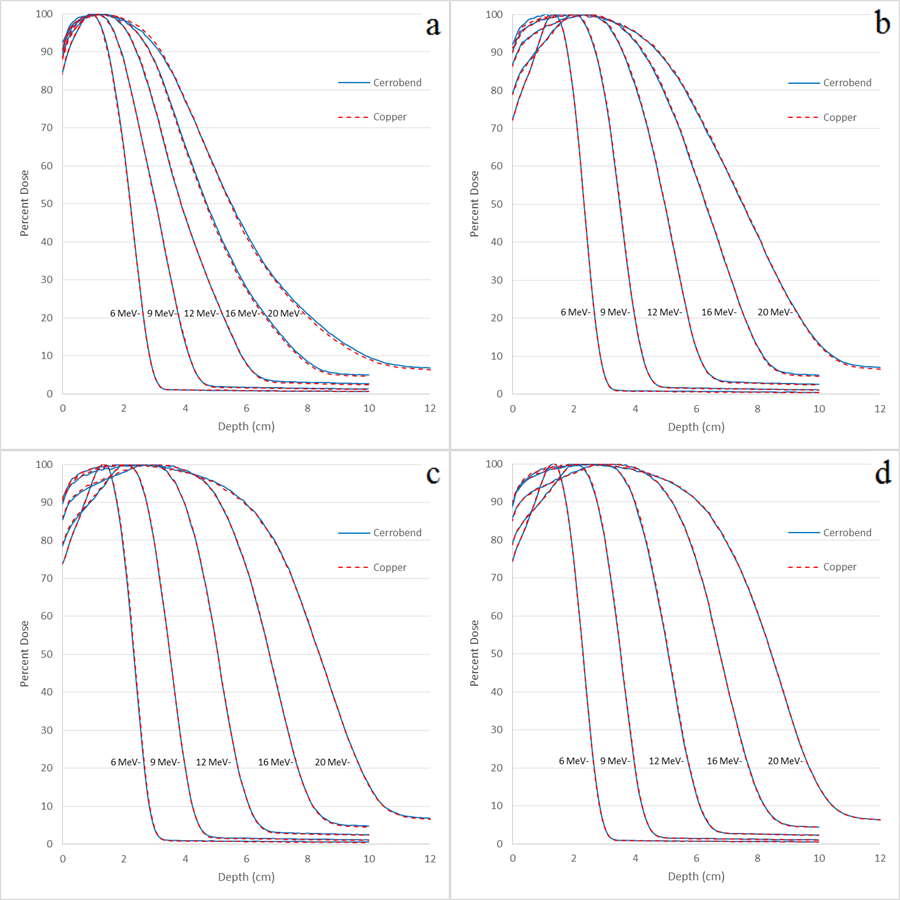


Figure 2: Comparison plots of PDDs for different field sizes in the 25x25 cm^2^ applicator at 100 cm SSD. Field sizes shown are (a) 2x2 cm^2^, (b) 4x4 cm^2^, (c) 12x12 cm^2^ and (d) 20x20 cm^2^

.


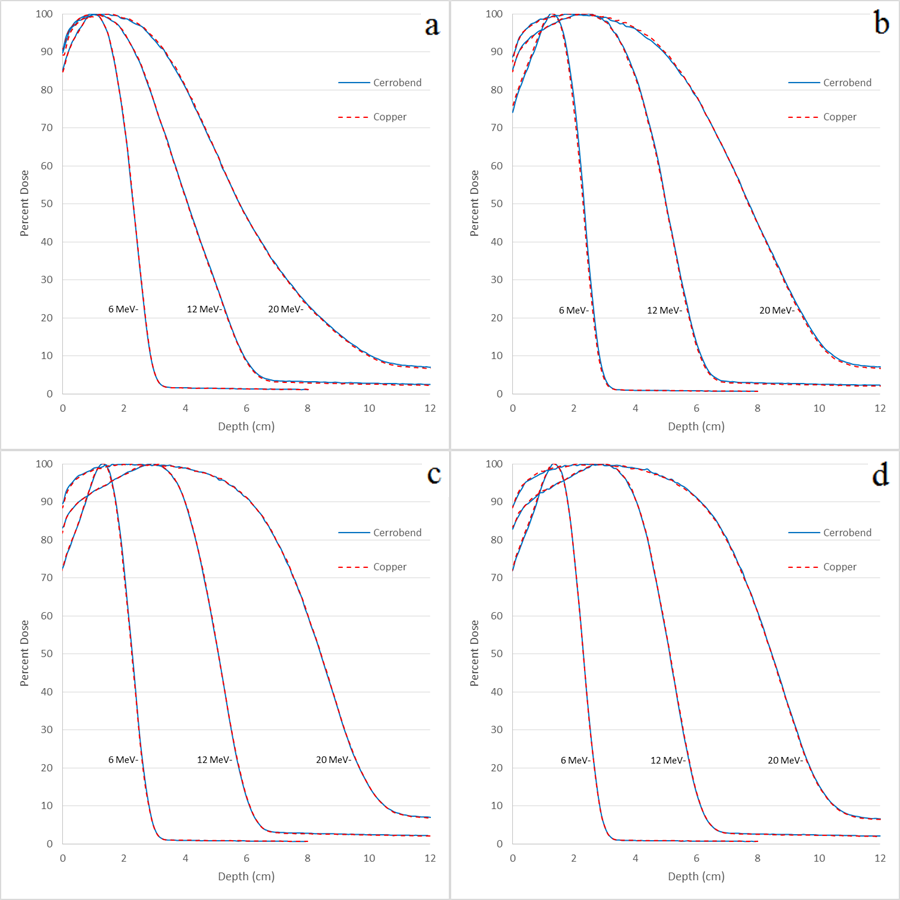


Figure 3: Comparison plots of PDDs for different field sizes in the 25x25 cm^2^ applicator at 110 cm SSD. Field sizes shown are (a) 2x2 cm^2^, (b) 4x4 cm^2^, (c) 12x12 cm^2^ and (d) 20x20 cm^2^

.


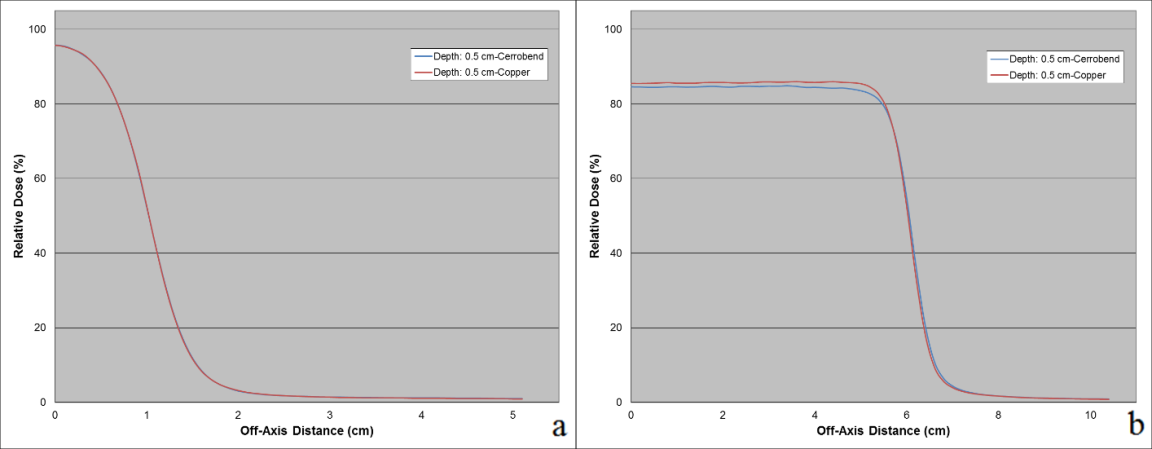

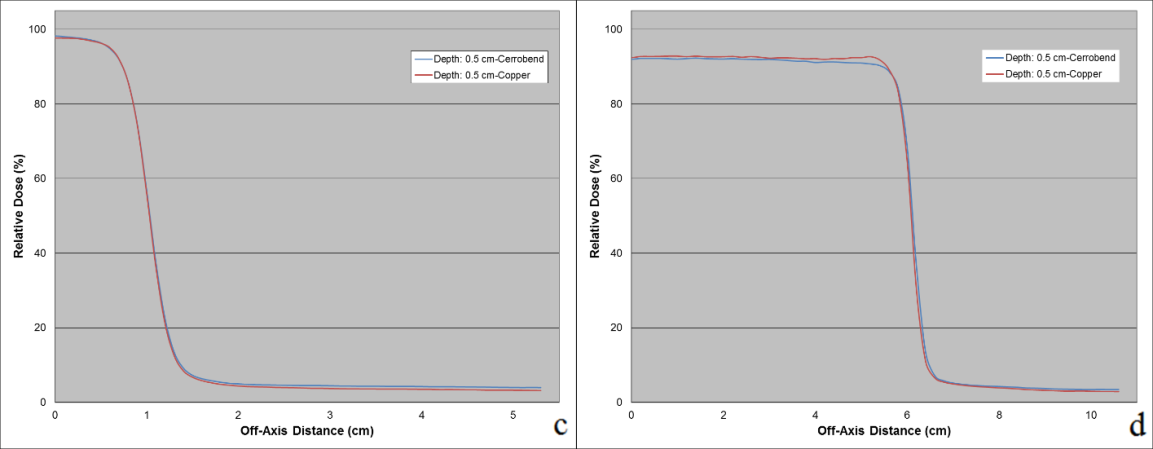

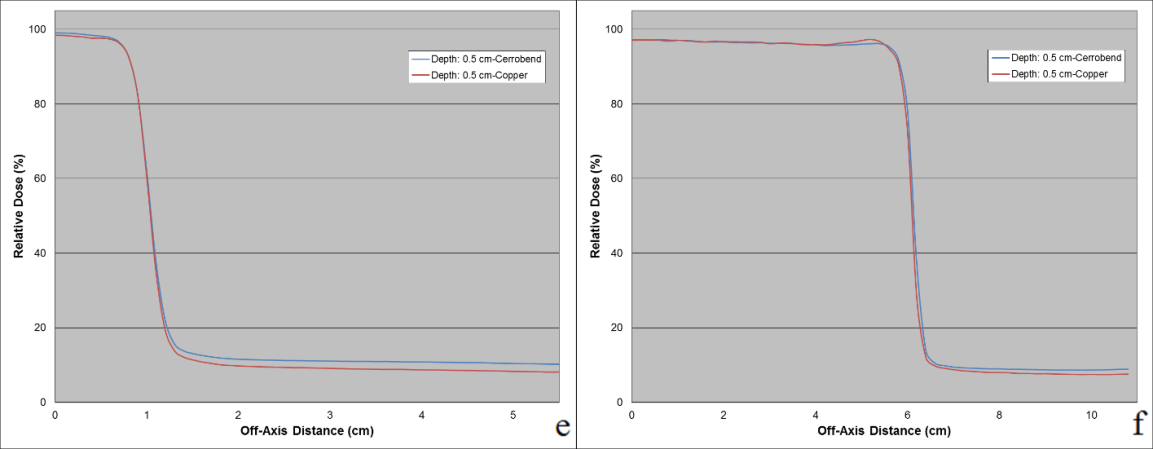


Figure 4: Off-axis relative dose profile measurements for copper and Cerrobend inserts of 2x2 cm^2^ field size (LEFT) and 12x12 cm^2^ field size (RIGHT) for the 25x25 cm^2^ applicator at 100 cm SSD for all three energies

. Energies of 6 MeV, 12 MeV, and 20 MeV are shown for the 2x2 cm^2^ field size (a, c, and e respectively) and the 12x12 cm^2^ field size (b, d, f respectively). Profiles are normalized to the central axis dose maxima.


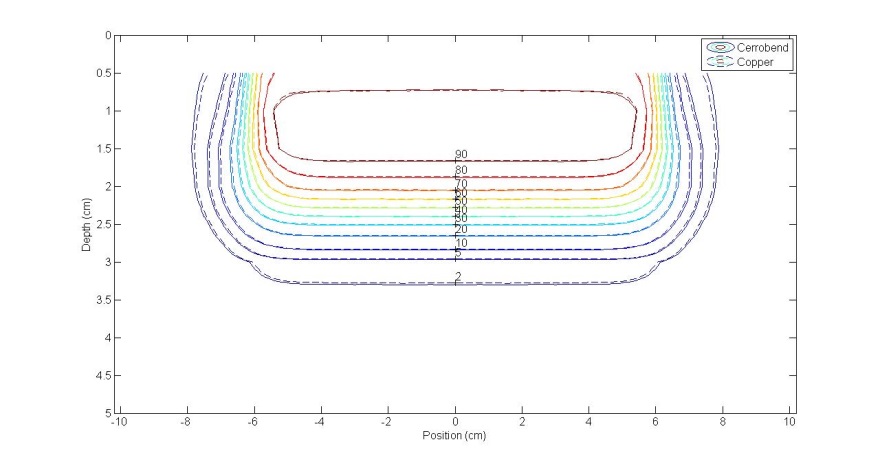


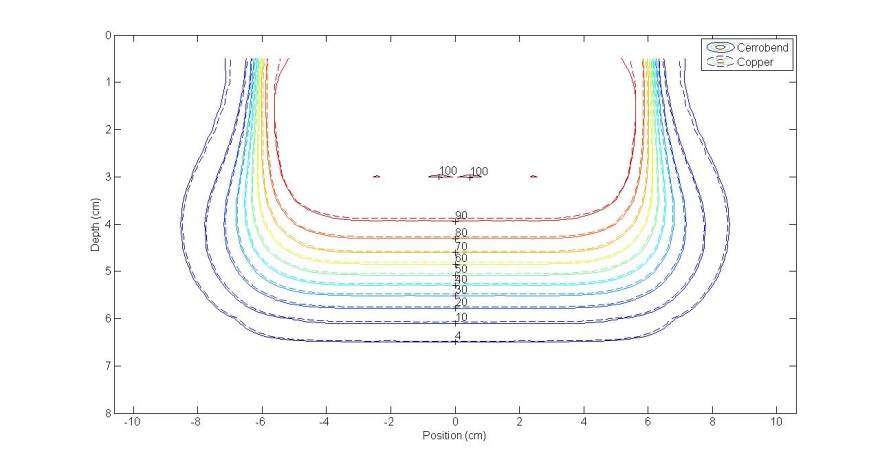


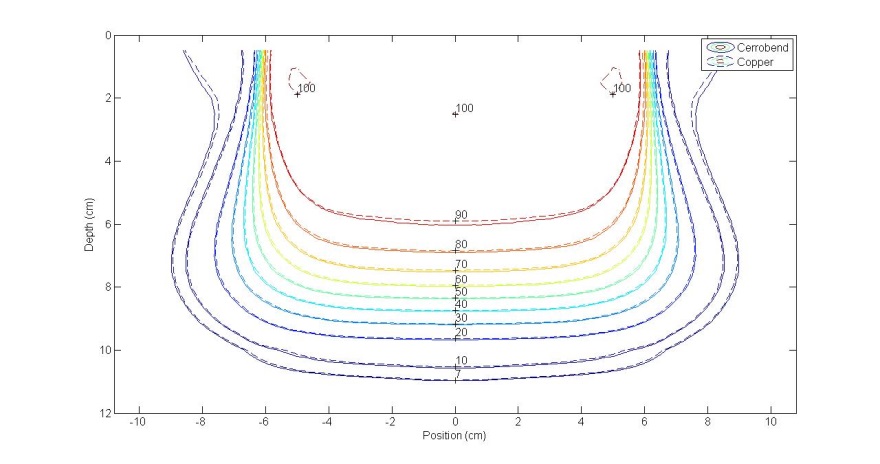


Figure 5: Absolute isodose comparison between Cerrobend (solid lines) and copper (dashed lines) for a 12x12 cm^2^ insert in a 15x15 cm^2^ applicator at (a) 6 MeV, (b) 12 MeV and (c) 20 MeV and 100 cm SSD

. All points passed the 2%/1mm criteria. The OCF was 1.000.


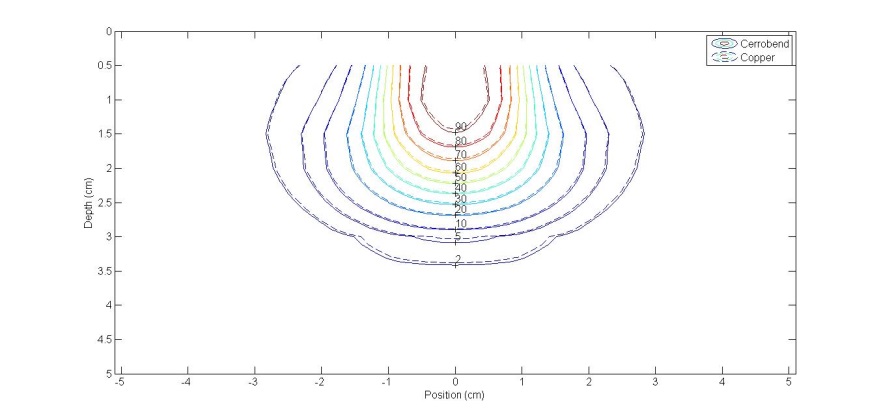

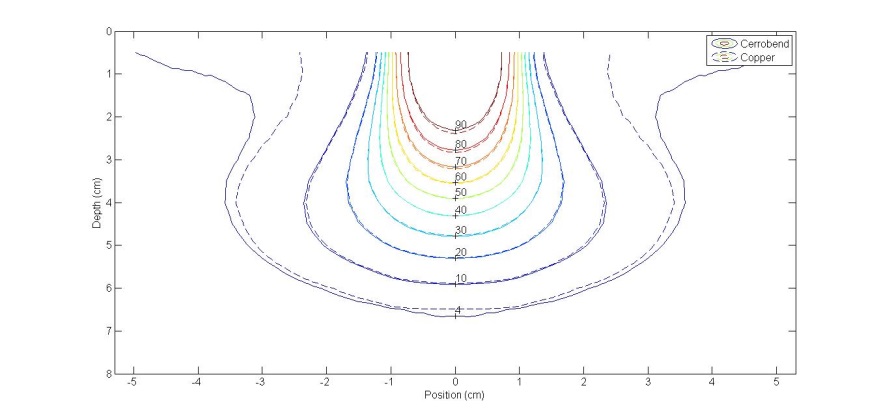

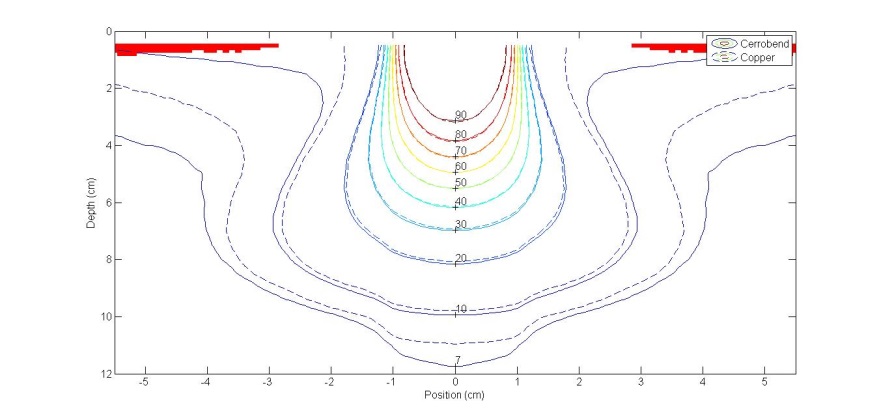


Figure 6: Absolute isodose comparison between Cerrobend (solid lines) and copper (dashed lines) for a 2x2 cm^2^ insert in a 25x25 cm^2^ applicator at (a) 6 MeV, (b) 12 MeV, and (c) 20 MeV and 100 cm SSD

. The red pixels on the isodose plot mark points which failed the 2%/1mm criteria (98.90% of points passed criteria). The OCF was 0.991.


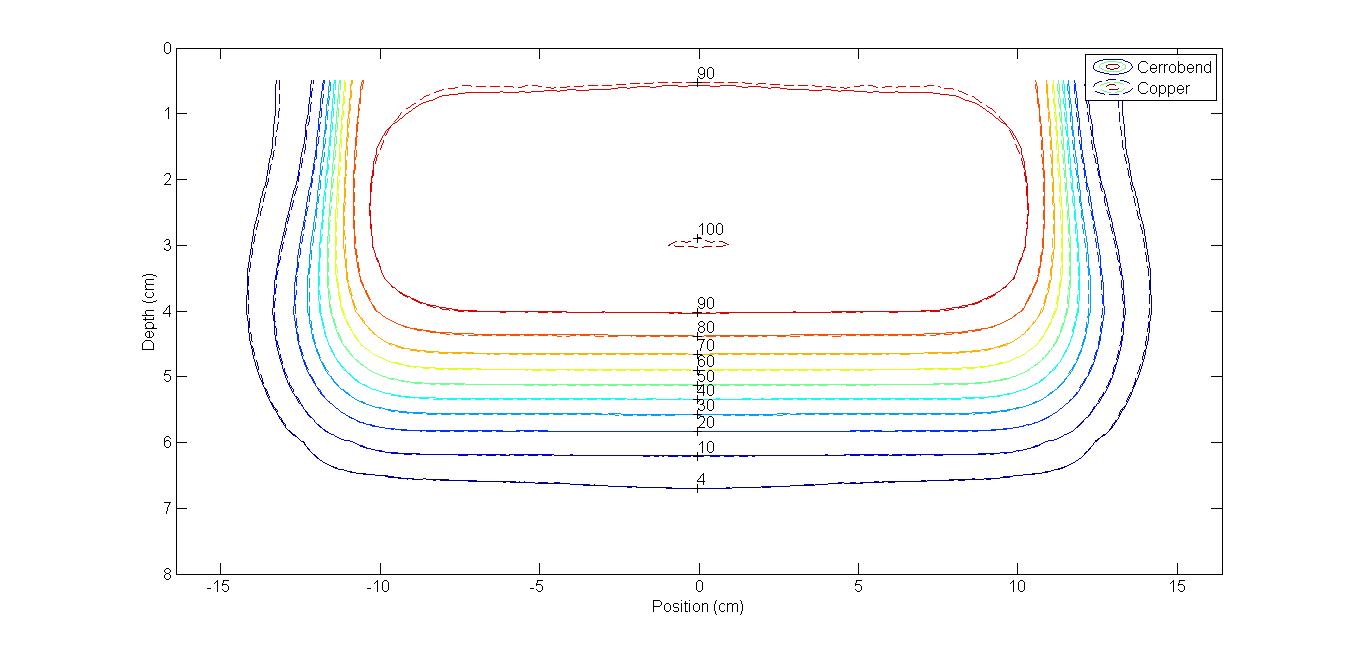


Figure 7: Absolute isodose comparison between Cerrobend (solid lines) and copper (dashed lines) for a 20x20 cm^2^ insert in a 25x25 cm^2^ applicator at 12 MeV and 110 cm SSD

. All points passed the 2%/1mm criteria. The OCF was 1.00.


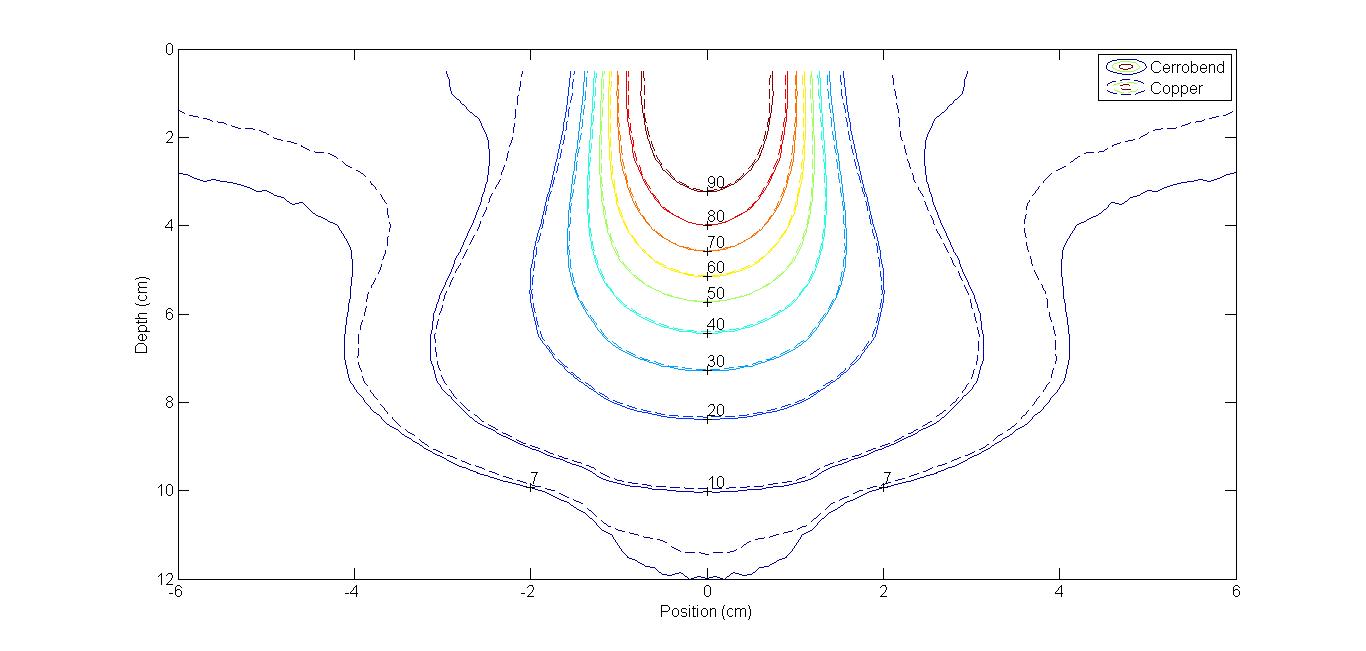


Figure 8: Absolute isodose comparison between Cerrobend (solid lines) and copper (dashed lines) for a 2x2 cm^2^ insert in a 25x25 cm^2^ applicator at 20 MeV and 110 cm SSD

. All points passed the 2%/1mm criteria. The OCF was 0.993.

**VII. Tables**

| Field Size (cm) | Applicator Size (cm) | | | | |
| --- | --- | --- | --- | --- | --- |
|  | **6x6** | **10x10** | **15x15** | **20x20** | **25x25** |
| 2x2 | X | X | X | X | X |
| 3x3 | X | X | X | X | X |
| 4x4 | X | X | X | X | X |
| 6x6 | N/A | X | X | X | X |
| 8x8 |  | X | X | X | X |
| 10x10 |  | N/A | X | X | X |
| 12x12 |  |  | X | X | X |
| 15x15 |  |  | N/A | X | X |
| 20x20 |  |  |  | N/A | X |

Table I: Summary of all insert field size-applicator combinations obtained for dosimetric comparisons.

A full set was obtained for both copper and Cerrobend. Open applicators, requiring no custom insert, were not compared.

| Field Size (cm) | Applicator Size (cm) | | | | |
| --- | --- | --- | --- | --- | --- |
|  | **6x6** | **10x10** | **15x15** | **20x20** | **25x25** |
| 2x2 | X |  | X |  | X |
| 3x3 | X |  |  |  | X |
| 4x4 | X |  | X |  | X |
| 6x6 |  |  |  |  | X |
| 8x8 |  |  | X |  | X |
| 10x10 |  |  |  |  | X |
| 12x12 |  |  | X |  | X |
| 15x15 |  |  |  |  | X |
| 20x20 |  |  |  |  | X |

Table II: Measurement subset

for off-axis relative dose profile measurements using energies 6, 12 and 20 MeV at 100 cm and 110 cm SSD. This measurement subset was also used for PDD and output measurements at 110 cm SSD.

| ∆D_x_ (%D_max_)  (Cerr- Cu) | 6 MeV | | | 12 MeV | | | 20 MeV | | |
| --- | --- | --- | --- | --- | --- | --- | --- | --- | --- |
| Field  Size (cm^2^) | Applicator (cm^2^) | | | Applicator (cm^2^) | | | Applicator (cm^2^) | | |
|  | 6x6 | 20x20 | 25x25 | 6x6 | 20x20 | 25x25 | 6x6 | 20x20 | 25x25 |
| 2x2 | 0.0 | 0.0 | 0.0 | 0.0 | 0.2 | 0.3 | 0.0 | 0.5 | 0.5 |
| 4x4 | -0.1 | 0.0 | 0.0 | 0.0 | 0.1 | 0.2 | 0.0 | 0.1 | 0.4 |
| 12x12 | N/A | 0.0 | 0.1 | N/A | 0.1 | 0.1 | N/A | 0.2 | 0.3 |

Table III: Central-axis photon dose difference (Cerrobend – Copper). Values presented are a percentage of the central-axis maximum dose. Generally, as the applicator size and energy increase the difference increases. In addition as the field size increases the difference decreases.

| Energy | Minimum OCF | Maximum OCF |
| --- | --- | --- |
| 6 MeV | 0.992 | 1.008 |
| 9 MeV | 0.992 | 1.006 |
| 12 MeV | 0.988 | 1.005 |
| 16 MeV | 0.983 | 1.005 |
| 20 MeV | 0.986 | 1.009 |
| All Energies | 0.983 | 1.009 |

Table IV: Minimum and maximum output correction factors at each energy and for all energies measured at 100 cm SSD

.

| OCF | 6 MeV | | | 12 MeV | | | 20 MeV | | |
| --- | --- | --- | --- | --- | --- | --- | --- | --- | --- |
| Field  Size (cm^2^) | Applicator (cm^2^) | | | Applicator (cm^2^) | | | Applicator (cm^2^) | | |
|  | 6x6 | 15x15 | 25x25 | 6x6 | 15x15 | 25x25 | 6x6 | 15x15 | 25x25 |
| 2x2 | 1.000 | 0.993 | 0.992 | 1.005 | 0.998 | 1.000 | 1.003 | 0.991 | 0.991 |
| 3x3 | 1.007 | 1.002 | 0.995 | 1.005 | 1.000 | 0.988 | 1.007 | 0.995 | 0.986 |
| 4x4 | 1.004 | 1.008 | 0.999 | 1.004 | 1.003 | 0.994 | 1.007 | 0.997 | 0.988 |
| 6x6 | N/A | 1.002 | 0.997 | N/A | 1.001 | 0.995 | N/A | 1.003 | 0.994 |
| 8x8 | N/A | 0.999 | 0.997 | N/A | 1.000 | 0.995 | N/A | 1.003 | 0.994 |
| 10x10 | N/A | 0.998 | 0.998 | N/A | 0.999 | 0.995 | N/A | 1.001 | 0.996 |
| 12x12 | N/A | 0.996 | 0.997 | N/A | 0.996 | 0.995 | N/A | 1.000 | 0.997 |
| 15x15 | N/A | N/A | 0.995 | N/A | N/A | 0.994 | N/A | N/A | 0.995 |
| 20x20 | N/A | N/A | 0.996 | N/A | N/A | 0.995 | N/A | N/A | 0.995 |

Table V: A subset of the OCFs covering the range of energies, field sizes, and applicators measured at 100 cm SSD.

| Energy | Minimum OCF | Maximum OCF |
| --- | --- | --- |
| 6 MeV | 0.990 | 1.006 |
| 12 MeV | 0.994 | 1.005 |
| 20 MeV | 0.990 | 1.003 |
| All Energies | 0.990 | 1.006 |

Table VI: Table IV: Minimum and maximum output correction factors at each energy and for all energies measured at 110 cm SSD

.

| OCF | 6 MeV | | | 12 MeV | | | 20 MeV | | |
| --- | --- | --- | --- | --- | --- | --- | --- | --- | --- |
| Field  Size (cm^2^) | Applicator (cm^2^) | | | Applicator (cm^2^) | | | Applicator (cm^2^) | | |
|  | 6x6 | 15x15 | 25x25 | 6x6 | 15x15 | 25x25 | 6x6 | 15x15 | 25x25 |
| 2x2 | 1.005 | 0.993 | 0.990 | 0.994 | 0.996 | 0.997 | 1.002 | 0.997 | 0.993 |
| 4x4 | 1.004 | 1.006 | 1.003 | 1.004 | 1.005 | 1.002 | 1.001 | 1.000 | 0.990 |
| 6x6 | N/A | N/A | 1.001 | N/A | N/A | 0.998 | N/A | N/A | 0.992 |
| 8x8 | N/A | 1.006 | 1.004 | N/A | 1.005 | 1.002 | N/A | 1.002 | 0.995 |
| 10x10 | N/A | N/A | 1.004 | N/A | N/A | 1.002 | N/A | N/A | 0.997 |
| 12x12 | N/A | 1.000 | 1.002 | N/A | 0.999 | 1.000 | N/A | 1.000 | 0.997 |
| 15x15 | N/A | N/A | 1.000 | N/A | N/A | 0.999 | N/A | N/A | 0.999 |
| 20x20 | N/A | N/A | 1.000 | N/A | N/A | 1.000 | N/A | N/A | 1.000 |

Table VII: A subset of the OCFs covering the range of energies, field sizes, and applicators measured at 110 cm SSD.
